# Selective Hydrogenation of Aldehydes Using a Well‐Defined Fe(II) PNP Pincer Complex in Biphasic Medium

**DOI:** 10.1002/cctc.201800841

**Published:** 2018-08-22

**Authors:** Stefan Weber, Julian Brünig, Veronika Zeindlhofer, Christian Schröder, Berthold Stöger, Andreas Limbeck, Karl Kirchner, Katharina Bica

**Affiliations:** ^1^ Institute of Applied Synthetic Chemistry Vienna University of Technology Getreidemarkt 9/163-AC Wien A-1060 Austria; ^2^ Department of Computational Biological Chemistry University of Vienna Faculty of Chemistry Währingerstrasse 17 Wien A-1090 Austria; ^3^ X-Ray Center Vienna University of Technology Getreidemarkt 9 Wien A-1060 Austria; ^4^ Institute of Chemical Technologies and Analytics Vienna University of Technology Getreidemarkt 9/163-AC Wien A-1060 Austria

**Keywords:** Hydrogenation, aldehydes iron, pincer complexes, ionic liquids, biphasic catalysis

## Abstract

A biphasic process for the hydrogenation of aldehydes was developed using a well‐defined iron (II) PNP pincer complex as model system to investigate the performance of various ionic liquids. A number of suitable hydrophobic ionic liquids based on the N(Tf)_2_^−^ anion were identified, allowing to immobilize the iron (II) catalyst in the ionic liquid layer and to facilitate the separation of the desired alcohols. Further studies showed that targeted Brønsted basic ionic liquids can eliminate the need of an external base to activate the catalyst.

## Introduction

The design and application of new catalysts and catalytic systems is a key driver of sustainable chemistry and a constant field of innovation. To date, the chemical industry ranging from bulk to fine chemical production relies heavily on the use of metal catalysts that are often based on rare noble metals as catalytically active site, particularly in case of homogenous catalysis. In the light of the limited abundance and the threat of a global shortage, the development of base metal catalysts relying on abundant and low cost metals with low toxicity has received increasing attention.^1^ Among all potential candidates, iron as the most common element in the earth's crust with its broad occurrence in biological systems is a particularly attractive candidate for the formation of well‐defined base metal catalysts.^2^ While iron‐based catalysts typically show a rather low reactivity compared to noble metals catalysts, the development of novel and finely tuned ligands systems in this rapidly expanding research field can compensate this issue. Nowadays, a broad variety of transformations, including the industrially important chemoselective reduction of polar double bonds such as aldehydes can be realized with high efficiency.^3^

**Figure 1:**
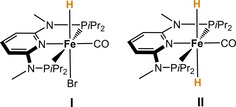
Pre‐catalyst [Fe(PNP^Me^‐*i*Pr)(CO)(H)(Br)] (**I**) and active species *trans*‐[Fe(PNP^Me^‐*i*Pr)(CO)(H)_2_)] (**II**) used for the biphasic reduction of aldehydes in these studies.

A milestone in the development of highly active iron‐based catalysts for the reduction of carbonyl groups was the implementation of pincer ligands, providing a basis for the mild and chemoselective iron‐catalyzed reduction of aldehydes, ketones, imines but also of CO_2_ under benign conditions and moderate hydrogen pressure.^4^ In 2014, Kirchner and co‐workers developed the iron (II) pincer complex [Fe(PNPMe‐*i*Pr)(CO)(H)(Br)] (**I**) that showed remarkable reactivity and chemoselectivity for the reduction of aldehydes.^5^ The first step of the catalytic cycle is the activation of pre‐catalyst **I** to form a mixture of *cis*‐ and *trans*‐dihydride complexes [Fe(PNPMe‐*i*Pr)(CO)(H)_2_] (**II**) upon addition of dihydrogen in the presence of base, with the *trans* isomer being the catalytically active species (Figure 1). Key step is the regeneration of the catalytically active complex **II** which requires dissociation of the product alkoxide to enable coordination and heterolytic cleavage of dihydrogen. Thus, high catalytic activity was observed in the protic solvent EtOH (relative polarity 0.654) which facilitates alkoxide dissociation and stabilization, while no conversion was observed in aprotic and low polarity solvents such as *n*‐heptane (0.009).^6^

As a result of the homogenous reaction environment in EtOH, difficulties in the separation of the formed alcohols and catalyst from the solvent ethanol arise as it is typical for homogenously catalyzed processes. To solve these issues, a number of strategies have been developed that can balance the positive aspects of homogenous and heterogeneous catalysis.^7^ Biphasic catalysis, in which the catalysts resides in one of the two phases while the product is dissolved in the second phase can facilitate problems encountered with separation but maintain the high activity associated with homogenous catalysis.^8^ This concept of liquid‐liquid biphasic catalysis is well established for different biphasic systems, i. e., aqueous‐organic solvent mixtures, and currently applied in several industrially relevant processes such as the Rhône‐Poulenc Ruhrchemie process for hydroformylation.^9^ More recently ionic liquids broadened the application range for liquid‐liquid biphasic catalysis, as they can avoid limitations of aqueous systems.^10^ Compatibility issues of organometallic species with water can be often overcome using ionic liquids instead. Moreover, many classical transition‐metal catalyst precursors are readily soluble in ionic liquids rendering the synthesis of specially designed ligands obsolete as it would be required for aqueous, fluorinated, or supercritical fluid‐based catalytic processes.^11^

We recently demonstrated that **I** can also be conveniently immobilized on precoated silica in a supported ionic liquid phase (SILP) approach using the hydrophobic ionic liquid [C_4_m_2_im]N(Tf)_2_.^12^ Reasonable high turnover frequencies (TOF) and turnover numbers (TON) could achieved with this catalyst system. For an extension of this approach to other SILP systems, i. e. other ionic liquids and other support materials, the wetting properties of a particular IL/support system play a key role. A strong affinity between the ionic liquid and the support surface is important both for the impregnation of the support with IL/catalyst solution, i. e. an even thin‐film coverage of the inner pore surface, and its resistance against IL/catalyst leaching. Bordes *et al*. have shown^13^ in a recent study of the wettability of graphite by ionic liquids, that the solid/liquid interfacial energy γ_SL_ is the best experimentally accessible parameter for the affinity of ionic liquids toward a solid surface: the lower γ_SL_, the lower the liquid contact angle and the higher the adhesion of a liquid film on a solid surface. Since γ_SL_ depends on both the solid surface energy γ_SV_ and the liquid surface tension γ_LV_ *via* Young‘s equation, the most suitable liquid with the strongest affinity must be found separately for each solid support material, i. e. each SILP system must be individually optimized and a pool of suitable ionic liquids must be available. For this purpose, we utilize here the iron (II) catalyzed reduction of aldehydes under biphasic conditions as model reaction and investigate reactivity, substrate dependence and catalyst recycling with a variety of different ionic liquids in order to create a pool of functional systems for their later application and optimization in SILP catalysis.

## Results and Discussion

**Figure 2:**
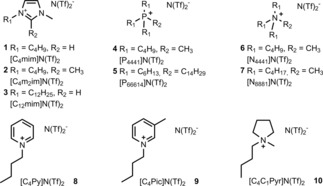
Hydrophobic ionic liquids used for the biphasic reduction of aldehydes.

A number of critical aspects need to be considered when selecting ionic liquids for the biphasic process, including a high solubility and stability of the pre‐catalyst and the active species in the ionic liquid, but also a low miscibility of ionic liquid and organic phase. Based on literature data and on our previous experience, we selected a set of hydrophobic ionic liquids based on the N(Tf)_2_^−^ anion for an initial screening. Apart from the favorable physical properties in terms of viscosity, the very weakly coordination nature of N(Tf)_2_^−^ should avoid ligand substitution reactions on the (pre‐) catalyst while also providing a higher hydrogen gas solubility compared to other hydrophilic ionic liquids with BF_4_^−^ or PF_6_^−^ anion.^14^ According to these considerations, a pool of ten hydrophobic ionic liquids with different cations and variable alkyl chain length was selected as shown in Figure 2.

**Scheme 1:**
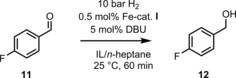
Catalytic reduction of 4‐fluorobenzaldehyde in ionic liquid/*n*‐heptane biphasic medium.

Initial investigations on the catalytic properties were performed with 4‐fluorobenzaldehyde as model system due to our previous research and its facile analysis *via* ^19^F{^1^H} NMR spectroscopy (Scheme 1). In order to minimize the required amount of ionic liquid, the biphasic system was composed of 1.5 ml of *n*‐heptane and 250 mg ionic liquid, which was sufficient to dissolve 0.5 mol % of the Fe‐PNP pre‐catalyst (S/C 200) used in all cases.

**Figure 3:**
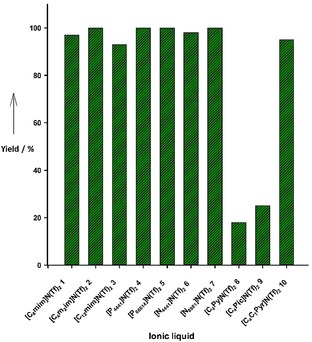
Results of the biphasic reduction of 4‐fluorobenzaldehyde with various hydrophobic ionic liquids.

To activate the pre‐catalyst **I**, DBU (1,8‐diaza‐bicyclo [5.4.0] undec‐7‐ene) (5 mol %) was added, and hydrogenations were routinely run at 10 bar hydrogen pressure for 60 min reaction time. With the exception of pyridinium and picolinium‐based ionic liquids **8** and **9**, all ionic liquids were suitable for the biphasic reduction of 4‐fluorobenzaldehyde, and the corresponding alcohol was formed as sole product in high yields (Figure 3).

In case of pyridinium and picolinium salts, drastically lower yields (<25 %) were observed presumably due to decomposition of the catalyst in these ionic liquids. While the ionic liquid layer typically had an orange‐red color after the reduction, pyridinium‐based systems rapidly turned black clearly suggesting an undesired interaction between the catalyst and these particular ionic liquid cations. Further analysis *via* NMR and GC‐MS did not reveal any evidence for alteration of the pyridinium and picolinium‐based ionic liquids indicating that an unintended reduction of the ionic liquids was not responsible for the failure of the reaction. It is interesting to note that there is little difference between the performance of imidazolium‐based cations [C_4_mim]N(Tf)_2_ **1** and [C_4_m_2_im]N(Tf)_2_ **2**. This suggests that the presence of an acidic proton in the C‐2 position and the ability to form an *N*‐heterocyclic carbene (NHC)‐ligand does not interfere with the reaction as it is often observed when reactive organometallic species are involved.^15^ The alkyl chain length had limited impact on the catalytic performance and comparable yields were found for the more viscous long alkyl chain ionic liquids [C_12_mim]N(Tf)_2_ **3**, [P_66614_]N(Tf)_2_ **5** and [N_8881_]N(Tf)_2_ **7** and the corresponding short chain derivatives [C_4_mim]N(Tf)_2_ **1**, [P_4441_]N(Tf)_2_ **4** and [N_4441_]N(Tf)_2_ **6**. However, from NMR analysis of the *n*‐heptane layer it was found that leaching of these more hydrophobic ionic liquids into the organic phase took place. Such a behavior was not encountered in case of the short‐chain derivatives.

Apart from undesired leaching of ionic liquid into the organic layer, the distribution of substrate, catalyst and product is a key aspect that will influence the efficiency of the reaction as well as separation of the products in biphasic catalysis. In general, the reduction of aldehydes is not an ideal situation for catalysis in organic/ionic liquid biphasic systems, as the more polar alcohol formed during the reactions is more likely to migrate to the ionic liquid phase. This unfavorable situation can be partially compensated when using a large volumetric excess of *n*‐heptane compared to ionic liquid. We further studied concentration effects of 4‐fluorobenzaldehyde **11** and 4‐fluorobenzylalcohol **12** in a biphasic model system composed of ionic liquid [P_4441_]N(Tf)_2_ and *n*‐heptane similar to the ratio and condition used for catalysis. These substrates were separately added to a biphasic model system composed of 1.5 ml of *n*‐heptane and 250 mg of ionic liquid [P_4441_]N(Tf)_2_ **4**, stirred for 60 min at 25 °C. ^19^F{^1^H} NMR measurements were carried out in the presence of an external standard (fluorobenzene) to quantify the distribution of substrate and product between the two phases. As expected, the less polar aldehyde **11** had a higher affinity for the organic phase compared to the product, and 0.62 mmol (corresponding to 31 %) 4‐fluorobenzaldehyde **11** were found in the *n*‐heptane layer. The more polar product 4‐fluorobenzylalcohol **12** is more likely to migrate to the ionic liquids phase, and only 0.32 mmol (16 %) were detected in the organic phase of the pure model system. However, the efficiency of the ionic liquid‐heptane biphasic system is evident when addressing the distribution of the Fe(II)‐PNP catalyst between ionic liquid and organic phase. ^31^P{^1^H} NMR analysis of *n*‐heptane could not detect any traces of catalyst. Moreover, ICP‐MS analysis revealed an iron content of <10 ng in the organic phase which clearly shows that no leaching of the catalyst into the *n*‐heptane solution takes place. Likewise, we did not detect DBU in the organic phase. It has to be noted that DBU is predominantly present in its protonated cationic form after activation of the pre‐catalyst **I** and is thus immobilized in the polar ionic liquid rather than in the apolar *n*‐heptane layer.

Due to the necessity of a base such as DBU during the iron‐catalyzed hydrogenation with Kirchner's catalyst, special attention was dedicated to Brønsted basic ionic liquids with dual function. In general, Brønsted basic ionic liquids contain at least one Brønsted basic group either on the cation or on the anion.^16^ Basic ionic liquids can be used as an alternative to commonly used (in) organic bases, and several applications in organic reactions such as esterifications,^17^ Michael additions^18^ or Aldol reactions^19^ have been reported. Non‐volatile organic salts with amine functionalities were also used to immobilize transition metal catalysts, for example palladium species^20^ or, more recently well‐defined ruthenium catalysts that have been employed for the elegant continuous flow hydrogenation of carbon dioxide to formic acid.^21^

**Figure 4:**
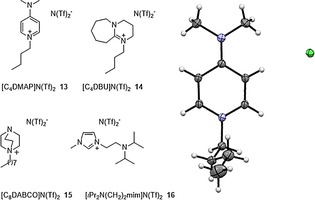
Brønsted‐basic ionic liquids for the biphasic reduction of aldehydes. The inset displays the X‐ray structure of the intermediate [C_4_DMAP]Cl (see ESI for more details).

We therefore expanded the pool of ionic liquids with four Brønsted basic ionic liquids to mimic typical organic bases (Figure 4). Ionic liquids with 4‐(dimethylamino) pyridine (DMAP), 1,8‐diazabicycloundecen‐7‐ene (DBU) and 1,4‐diazabicyclo‐[2.2.2] octane (DABCO) structural motif (**13**–**15**) were prepared *via* selective alkylation of diamines and successive ion exchange. In case of the DMAP‐based ionic liquid **13**, crystals were grown from the intermediated chloride salt that supported its structure. This set was complemented with an amino‐functionalized imidazolium derivative as *N*,*N*‐diisopropylethylamine (DIPEA) analogue (**16**) that has been previous used for palladium catalyzed cross coupling reactions. An interesting pattern was observed when studying the catalytic activity of [Fe(PNPMe‐*i*Pr)(CO)(H)(Br)] (**I**) in Brønsted basic ionic liquids **13**–**16** without additional base. The DIPEA and DABCO‐based ionic liquids were not suitable and failed, indicating that the pre‐catalyst was not activated in these ionic liquids (Table 1, entries 3–4). In contrast, excellent yields of 95 and 97 % were found with DBU and DMAP analogue in a biphasic *n*‐heptane/ionic liquid system, thereby eliminating the necessity of base addition in this reaction (Table 1, entries 1–2).

**Table 1:** Yields and kinetic data for the biphasic reduction of 4‐fluorobenzaldehyde in selected conventional and Brønsted‐basic ionic liquids.

Entry	Ionic liquid^[a]^	Yield [%]^[a]^	TOF [h^−1^]^[b]^	TON^[c])^
1	[C_4_DMAP]N(Tf)_2_ **13**	98	800	220
2	[C_4_DBU]N(Tf)_2_ **14**	97	480	240
3	[C_8_DABCO]N(Tf)_2_ **15**	<1	n.d.	n.d.
4	[*i*Pr_2_N(CH_2_)_2_mim]N(Tf)_2_ **16**	<1	n.d.	n.d.
5	[P_4441_]N(Tf)_2_ **4** ^[d]^	>99	1332	793
6	[C_4_m_2_im]N(Tf)_2_ **2** ^[b]^	>99	1008	1258

[a] Performed with 2 mmol aldehyde and 0.5 mol % pre‐catalyst in 250 mg ionic liquid/1.5 ml *n*‐heptane for 60 min. Yield determined *via* ^19^F NMR spectroscopy of organic and ionic liquid phase using fluorobenzene as external standard; [b] performed with 2 mmol aldehyde and 0.5 mol % pre‐catalyst in 250 mg ionic liquid/1.5 ml *n*‐heptane. TOF reported as amount of substrate [mmol]/(amount of catalyst [mmol]×reaction time for full conversion [h]); [c] performed with 20 mmol aldehyde and 0.05 mol % pre‐catalyst in 250 mg ionic liquid/1.5 ml *n*‐heptane for 80 h. Maximum TON reported as amount of product [mmol]/amount of catalyst [mmol]; [d] 5 mol % DBU added.

Further studies on the catalytic performance were performed with four selected ionic liquids, including [C_4_m_2_im]N(Tf)_2_ **2** and [P_4441_]N(Tf)_2_ **4** that gave the highest yields as well as the Brønsted basic ionic liquids **13** and **14**. The highest TOF was found for the phosphonium‐based ionic liquid [P_4441_]N(Tf)_2_ **4**, and complete conversion was observed in only 9 min reaction time. In case of the imidazolium‐based ionic liquid [C_4_m_2_im]N(Tf)_2_ **2** slightly longer reaction times of 12 minutes were required; however, the maximum TON was higher (Table 1, entries 5–6). In contrast, considerably lower TOF and TON values were determined for Brønsted‐basic ionic liquids [C_4_DBU]N(Tf)_2_ **13** and [C_4_DMAP]N(Tf)_2_ **14** (Table 1, entries 1–2).

**Figure 5:**
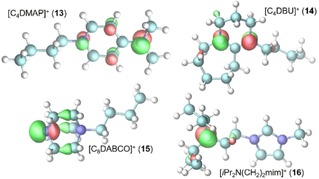
Molecular orbital description of the non‐binding HOMO orbitals of the alkaline ionic liquid cations. The bold numbers represent the corresponding N(Tf)_2_‐based ionic liquids.

The lower catalytic activity might be a result of a reduced electron density on the free nitrogen in the Brønsted‐basic ionic liquid cations (see Figure 5) compared to the free base. Although the actual values of the partial charge q_N_ in Table 2 are generally not an ideal measure for the basicity of the nitrogen atoms,^22^,^23^ relative trends for the basicity can still be observed when altering moieties of the compound. Except for the isopropyl amine compounds the charge density of the alkaline nitrogen is reduced by roughly 0.15e in the cationic compound compared to the pure base which probably corresponds to lower basicities of the charged compounds. This fact is confirmed by the respective proton affinities (PA) in Table 2. Lower proton affinities are usually correlated with lower basicity of the nitrogen atoms.^23^,^24^

**Table 2:** Computational analysis of the basicity of the nitrogen atoms of the cations and the corresponding bases. The bold numbers represent the corresponding N(Tf)_2_‐based ionic liquid.

cation/base	q_N_ [e]	α_N_ [Å^3^]	PA [eV]	SASA [Å^2^]
[C_4_DMAP]^+^ (**13**)	−0.147	1.66	10.30	2.0
DMAP	−0.323	1.51	11.21	1.5
[C_4_DBU]^+^ (**14**)	−0.213	1.19	9.54	0.7
DBU	−0.396	1.19	11.07	0.7
[C_8_DABCO]^+^ (**15**)	−0.507	1.11	11.06	11.8
DABCO	−0.635	1.22	11.97	11.4
[*i*Pr_2_N(CH_2_)_2_mim]^+^ (**16**)	−0.840	1.57	11.50	0.0
DIPEA	−0.836	1.50	12.06	0.0

However, arguments solely based on the Brønsted basicity cannot explain the failure of the compounds **15** and **16** as the alkaline nitrogen atoms in these compounds should have a higher basicity compared to those in **13** and **14**. The flexibility of the electron cloud at the alkaline nitrogen is characterized by its polarizability α_N_. Consequently, the ability to donate the lone pair to a Lewis electron acceptor should scale with the atomic polarizability on the nitrogen. Unfortunately, the nitrogen polarizabilities in Table 2 show no clear trend.

The majority of Lewis acid‐base reaction involve n−σ* or n−π* molecular orbital interactions.^22^ The corresponding non‐bonding HOMO of the alkaline ionic liquid cations are depicted in Figure 5. In contrast to the other cations, the n‐orbital of [C_8_DABCO]^+^ has a deformed shape for a non‐bonding molecular orbital which may indicate less effective interaction with the σ* or π* molecular orbital of the Lewis acid and thereby reducing the obtained yield in Table 2.

In addition to the electrostatic and energetic properties as well as the molecular orbitals discussed so far, steric reasons may also play a role for the interaction of the catalyst with the base. As visible from the solvent accessible surface (SASA) of the alkaline nitrogen in Table 2 the nitrogen of [C_4_DMAP]^+^ is more accessible compared to [C_4_DBU]^+^ which may explain the higher TOF and TON values in Table 1. In contrast, the alkaline nitrogen atom in [*i*Pr_2_N(CH_2_)_2_mim]^+^ and DIPEA seems to be sterically more hindered, thereby prohibiting any contact of the nitrogen and the catalyst.

**Figure 6:**
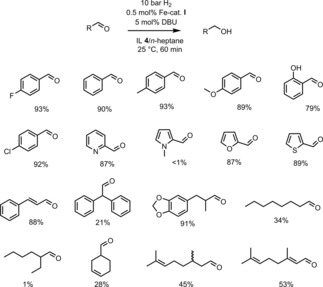
Substrate scope and limitation for a set of aromatic and aliphatic aldehydes. The reported yields refer to isolated yields of the pure corresponding alcohols.

After identifying the most suitable ideal ionic liquid for the biphasic reaction set‐up, we addressed substrate scope and application range for a set of (hetero‐)aromatic and aliphatic aldehydes (Figure 6). To separate product and catalyst, the heptane phase was simply removed *via* decantation. Although the product was obtained in high purity without traces of starting material, catalyst, base or ionic liquid, evaporation of *n*‐heptane gave the product 4‐fluorobenzylalcohol **12** only in poor yield of 12 %. To increase the yield, the catalyst containing phase was successively extracted with diethyl ether to collect any remaining product. This resulted in an improved isolated yield of 93 %. While we did not observe any leaching of the iron‐based catalysts due to its extremely low solubility in diethyl ether, the increased solubility of phosphonium‐based ionic liquid [P_4441_]N(Tf)_2_ **4** resulted in trace contamination of the diethyl ether extract. Consequently, an additional filtration step over a batch of silica was performed to remove traces of ionic liquids and to obtain a pure product. This extraction with diethyl ether was routinely performed in the substrate screening to provide reliable results, since all products and ‐ in case of uncomplete reaction – aldehyde starting materials could be extracted with diethyl ether.

In general, good to excellent yields were achieved for aromatic systems, even in the presence of coordinating groups. Similar results were found for the heterocyclic substrates pyridine‐2‐carboxaldehyde, furfural and thiophene‐2‐carboxaldehyde with high isolated yields, whereas the reduction failed with *N*‐methyl‐pyrrol‐2‐carboxaldehyde aldehyde. This is in accordance with our previous observations using ethanol as solvent, indicating an unfavorable interaction between the Fe(II) PNP catalyst and pyrrole‐based heterocycles. The reduction of aliphatic aldehydes was more challenging and gave variable results. While the branched long‐chain aliphatic aldehyde 2‐ethylhexanal was not reduced, moderate yields were observed with octanal. Moderate to high yields were obtained for selected unsaturated aldehydes. It is particularly worth noticing that the chemoselective behavior for the reduction of aldehydes was preserved in the biphasic set‐up, since unsaturated aldehydes such as the challenging α,β‐unsaturated substrates cinnamic aldehyde and citral were selectively reduced to unsaturated alcohols that are of particular importance for the flavor and fragrance industries.

**Figure 7:**
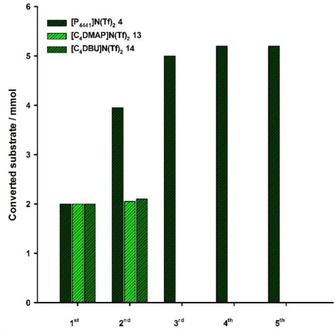
Cumulative turnover of 4‐fluorobenzaldehyde in biphasic reaction media using [P_4441_]N(Tf)_2_ 4, [C_4_DMAP]N(Tf)_2_ 13 or [C_4_DBU]N(Tf)_2_ 14 to immobilize the iron‐based pre‐catalyst [Fe(PNP^Me^‐*i*Pr)(CO)(H)(Br)] (I). Performed with 2 mmol aldehyde each run and 0.5 mol % pre‐catalyst in 250 mg ionic liquid/1.5 ml *n*‐heptane.

Apart from the benefits of simple product separation, liquid‐liquid biphasic catalysis also offers a powerful tool for catalyst recovery and recycling. However, initial attempts to recover the Fe(II) PNP catalyst after product extraction failed. Independent of the ionic liquid, we did not observe any conversion when fresh starting material was added. Further studies on long‐term stability of the catalyst in ionic liquids showed that the activated dihydride species is considerable less stable (see ESI Figure 1). ^31^P{^1^H} NMR spectroscopy indicated the rapid formation of several intractable decomposition products when the pre‐formed dihydride complex was kept in [P_4441_]N(Tf)_2_ **4**. This is a considerable difference and drawback compared to pre‐catalyst **I**, which is stable in [P_4441_]N(Tf)_2_ **4** even for one week according to ^31^P{^1^H} NMR spectroscopy. It was however possible to show a certain reusability of the catalyst immobilized in [P_4441_]N(Tf)_2_ **4** *via* a semi‐continuous addition of substrate (Figure 7). After 10 min reaction time a fresh batch of 4‐fluorobenzaldehyde (2 mmol) was added to prevent decomposition of the active. With this strategy, the catalyst remained active for four consecutive runs and a total of 5.2 mmol starting material can be converted. Similar experiments with the Brønsted‐basic ionic liquids [C_4_DMAP]N(Tf)_2_ **13** and [C_4_DBU]N(Tf)_2_ **14** were less successful as the catalytic activity ceased already after the first run.

## Conclusions

In summary, we showed that hydrophobic ionic liquids in combination with *n*‐heptane can be used as biphasic reaction media for the iron (II) catalyzed hydrogenation of aldehydes. The presented liquid‐liquid biphasic process presents a novel and improved methodology compared to the homogenous reaction in terms of product isolation and catalyst separation.

The iron (II) PNP pincer complex catalyst was efficiently immobilized in the ionic liquid phase without requiring any derivatization of the ligand, and no leaching in the organic phase could be detected. Hydrogenation of a set of aliphatic and aromatic aldehydes including unsaturated species was performed under comparatively mild conditions (25 °C, 1 h, 10 bar H_2_). However, while the products were typically obtained in high purities without contamination from catalyst, ligand or base, the necessity of an addition extractions step with diethyl ether in order to obtain high isolated yields of the formed alcohols reduction the utility of the biphasic system for this particular application. Clearly, a reduced amount of ionic liquid as used in our previous study with supported ionic liquid phases (SILPs) is beneficial and can overcome this issue.^12^ As for the use of supported catalysts, the facile separation of the catalyst containing ionic liquid allowed for a certain recovery and reuse of the catalyst for three runs, although losses in the catalytic activity due to partial decomposition of the Fe(II) catalyst in the absence of hydrogen could not be avoided. Eventually, the design of targeted Brønsted basic ionic liquids can eliminate the necessity of base addition and facilitate the reaction set‐up towards the future design of continuous flow processes.

## **Experimental Section**

All used reagents and solvents were purchased from commercial suppliers and directly used without further purification, if not stated otherwise. Anhydrous CH_2_Cl_2_, Et_2_O, *n*‐heptane, MeOH, THF and toluene were dried over molecular sieve and/or *via* Na/K alloy and degassed via pump freezing. ^1^H, ^19^F{^1^H} and ^31^P{^1^H} NMR were recorded in acetonitrile‐d_6_, chloroform‐d, methylene chloride‐d_2_, dimethylsulfoxide‐d_6_ or methanol‐d_4_ solution on a Bruker Avance 200 (200 MHz) or Bruker Avance 250 (250 MHz). All chemical shifts (δ) are reported in ppm, using tetramethylsilane for ^1^H, trichlorofluoromethane for ^19^F and H_3_PO_4_ (85 %) for ^31^P NMR spectra. All coupling constants (J) are reported in Hertz (Hz).

Determination of Fe concentrations was done using an inductively coupled plasma (ICP) optical emission spectrometer PerkinElmer OPTIMA 8300 equipped with an SC‐2 DX FAST sample preparation system. A customized single‐element (Merck, Roth) standard was used for the calibration.

X‐ray diffraction data of [C_4_DMAP]Cl⋅CHCl_3_ [CCDC entry 1825413] were collected at *T*=100 K in a dry stream of nitrogen on a Bruker Kappa APEX II diffractometer system using graphite‐monochromatized Mo‐*K*α radiation (λ=0.71073 Å) and fine sliced *ϕ*‐ and *ω**‐***scans. Data were reduced to intensity values with SAINT and an absorption correction was applied with the multi‐scan approach implemented in SADABS.^25^ The structure was solved by the dual space method implemented in SHELXT^26^ and refined against *F* ^2^ with JANA2006.^27^ Non‐hydrogen atoms were refined anisotropically. H atoms were placed in calculated positions and thereafter refined as riding on the parent C atoms. A chloroform solvent molecule was modelled as disordered about three positions. Molecular graphics were generated with the program MERCURY.^28^ Crystal data and experimental details are given in Table S1 in ESI.

Quantum‐chemical geometries of the compounds were optimized with GAUSSIAN09^24^ using B3LYP/6‐311++G(2d,2p) and a polarizable continuum model (PCM) with the dielectric constants $\epsilon \left(0\right)$ =14.0 and $\epsilon \left(\infty \right)$ =2.1 mimicking solvation in the ionic liquids [C_4_m_2_im]N(Tf)_2_ or [C_4_mim]N(Tf)_2_. Partial charges were determined by chelpg^23^ using ωB97XD/aug‐cc‐pVTZ/PCM to account for polarizable and dispersion effects. Atomic polarizabilities were obtained by applying a electric field of 0.0008 au in positive and negative x‐,y‐ and z‐direction using M06‐2X/Sadlej.^29^ Computational proton affinities (PA )^29^,^30^,^31^,^32^,^33^

(1) $$PA=\left(E\left(A\right)+ZPE\left(A\right)\right)-\left(E\left(HA^{+}\right)+ZPE\left(HA^{+}\right)\right)$$

were evaluated from the difference of the total energy of the protonated species HA^+^ and the neutral species A. Since the protonation adds additional vibrational degrees of freedom, both total energies were corrected for their respective zero‐point vibrational energy (ZPE ). The molecular orbitals were visualized using VMD^34^ with an iso value of 0.08. The solvent accessible surface (SASA) was calculated by VMD using a probe radius of 1.4 Å.

All ionic liquids **1**–**10** were synthesized according to standard methodologies, and analytical data was in accordance with literature. The ionic liquids were dried for at least 48 h with stirring at 10^−2^ bar and 50 °C before use and stored under argon atmosphere in a glove box. The water content was measured *via* Karl‐Fischer titration and was typically below <500 ppm.

For Brønsted basic ionic liquids **13**–**15** a modified protocol relying on selective alkylation of diamines and successive ion exchange was performed as indicated below.

### Synthesis of 1‐butyl‐4‐(dimethylamino) pyridine‐1‐ium Chloride ([C_4_DMAP]Cl)

*N,N*‐Dimethylpyridine‐4‐amine (11.4 g, 93 mmol) was dissolved in anhydrous acetonitrile and freshly distilled 1‐chlorobutane (16.9 g, 180 mmol) was added in one batch. The reaction mixture was refluxed for four days. During the reaction, the product precipitated from the reaction mixture as colorless solid. The solid was separated from the solution *via* filtration, washed three times with diethylether and dried under vacuum to yield [C_4_DMAP]Cl as colorless solid (19.2 g, 96 %). ^1^H NMR (200 MHz, methylene chloride‐d_2_) δ=8.44 (d, J=7.8 Hz, 2H), 6.92 (d, J=7.8 Hz, 2H), 4.27 (t, J=7.3 Hz, 2H), 3.16 (s, 6H), 1.77 (q, J=7.5 Hz, 2H), 1.39–1.18 (m, 2H), 0.88 ppm (t, J=7.3 Hz, 3H).

### Synthesis of 1‐butyl‐4‐(dimethylamino) pyridin‐1‐ium bis (trifluoromethane) Sulfonylimide ([C_4_DMAP]N(Tf)_2_) 13

[C_4_DMAP]Cl (7.0 g, 32.7 mmol) was dissolved in H_2_O and lithium bis (trifluoromethane) sulfonylimide (10.3 g, 35.9 mmol) in H_2_O was added dropwise. The mixture was stirred for one hour at room temperature. The biphasic reaction mixture was extracted three times with CH_2_Cl_2_. The combined organic phases were washed repeatedly with MilliQ‐grade H_2_O until no more chloride anions could be detected. The organic phase was dried over Na_2_SO_4_, filtrated and the solvent was removed. Remaining volatile traces were removed under high vacuum with stirring at 50 °C to yield [C_4_DMAP]N(Tf)_2_ **13** as colorless liquid (12.9 g, 86 %). ^1^H NMR (200 MHz, methylene chloride‐d_2_) δ=8.44 (d, J=7.8 Hz, 2H), 6.92 (d, J=7.8 Hz, 2H), 4.27 (t, J=7.3 Hz, 2H), 3.16 (s, 6H), 1.77 (q, J=7.5 Hz, 2H), 1.39–1.18 (m, 2H), 0.88 ppm (t, J=7.3 Hz, 3H).

### Synthesis of 1‐butyl‐2,3,4,6,7,8,9,10‐octahydro‐pyrimido [1,2‐a] azepin‐1‐ium chloride [C_4_DBU]Cl

Freshly distilled 2,3,4,6,7,8,9,10‐octahydropyrimido [1,2‐a] azepine (12.4 g, 82.0 mmol) was dissolved in anhydrous acetonitrile and freshly distilled 1‐chlorobutane (13.8 g, 152.0 mmol) was added dropwise. The reaction mixture was refluxed for four days to reach full conversion as detected by ^1^H NMR. The solvent was removed and the remaining orange oil was washed repeatedly with diethyl ether and ethyl acetate. Remaining volatiles were removed under high vacuum to yield [C_4_DBU]Cl as orange oil (11.1 g, 56 %). ^1^H‐NMR (250 MHz, chloroform‐d) δ=3.71–3.37 (m, 8H), 2.88–2.75 (m, 2H), 2.13 (t, J=5.9 Hz, 2H), 1.91–1.69 (m, 8H), 1.62 (quint, J=15.5, 7.5 Hz, 2H), 1.37 (sext, J=7.3 Hz, 2H), 0.98 ppm (t, J=7.2 Hz, 3H).

### Synthesis of 1‐butyl‐2,3,4,6,7,8,9,10‐octahydro‐pyrimido [1,2‐a] azepin‐1‐ium bis (trifluoromethane)‐sulfonylimide ([C_4_DBU]N(Tf)_2_) 14

[C_4_DBU]Cl (11.1 g, 45.3 mmol) was dissolved in H_2_O and lithium bis (trifluoromethane) sulfonylimide (14.3 g, 49.9 mmol) in H_2_O was added dropwise. The mixture was stirred for one hour at room temperature. The biphasic reaction mixture was extracted three times with CH_2_Cl_2_. The combined organic phases were washed repeatedly with MilliQ‐grade H_2_O until no more chloride anions could be detected. The organic phase was dried over Na_2_SO_4_, filtrated and the solvent was removed. Remaining volatile traces were removed under high vacuum with stirring at 50 °C to yield [C_4_DBU]N(Tf)_2_ **14** as orange liquid (18.8 g, 85 %). ^1^H NMR (250 MHz, chloroform‐d) δ=3.71–3.37 (m, 8H), 2.88–2.75 (m, 2H), 2.13 (t, J=5.9 Hz, 2H), 1.91–1.69 (m, 8H), 1.62 (quint, J=15.5, 7.5 Hz, 2H, ), 1.37 (sext, J=7.3 Hz, 2H), 0.98 ppm (t, J=7.2 Hz, 3H).

### Synthesis of 1‐octyl‐1,4‐diazabicyclo [2.2.2] octan‐1‐ium Bromide [C_8_DABCO]Br

1,4‐Diazabicyclo [2.2.2] octane (5.5 g, 49.1 mmol) was dissolved in anhydrous ethyl acetate and 1‐bromooctane (9.5 g, 49.1 mmol) dissolved in ethyl acetate was added dropwise. The reaction mixture was refluxed for three days. During the reaction, the product precipitated from the reaction mixture as light yellow solid. The solid was removed *via* filtration, washed several times with ethyl acetate and anhydrous tetrahydrofuran and dried under vacuum to yield [C_8_DABCO]Br as light yellow solid (5.9 g, 39 %). ^1^H NMR (250 MHz methylene chloride‐d_2_) δ=3.36–3.09 (m, 14H), 1.8–1.68 (m, 2H), 1.46–1.20 (m, 13H), 0.98 ppm (t, J=7.1 Hz, 3H).

### Synthesis of 1‐octyl‐1,4‐diazabicyclo [2.2.2] octan‐1‐ium bis (trifluoromethane) Sulfonylimide ([C_8_DABCO]N(Tf)_2_) 15

[C_8_DABCO]Br (4.88 g, 16 mmol) as dissolved in H_2_O and lithium bis (trifluoromethane) sulfonylimide (4.82 g, 16.8 mmol) in H_2_O was added dropwise. The mixture was stirred for one hour at room temperature. The biphasic reaction mixture was extracted three times with CH_2_Cl_2_. The combined organic phases were washed repeatedly with MilliQ‐grade H_2_O until no chloride anions could be detected. The organic phase was dried over Na_2_SO_4_, filtrated and the solvent was removed. Remaining volatile traces were removed under high vacuum with stirring at 50 °C to yield [C_8_DABCO]N(Tf)_2_ **14** as light yellow liquid (7.44 g, 92 %). ^1^H NMR (250 MHz methylene chloride‐d_2_) δ=3.36–3.09 (m, 14H), 1.8‐1.68 (m, 2H), 1.46–1.20 (m, 13H), 0.98 ppm (t, J=7.1 Hz, 3H).

### Synthesis of 1‐(2‐(diisopropylamino) ethyl)‐3‐methyl‐imidazolium Chloride

A round bottom flask was charged with 2‐(diisopropylamino) ethylchloride hydrochloride (14.01 g, 70 mmol) and 1‐methylimidazol (8.0 g, 97 mmol). The mixture was suspended in anhydrous ethanol and refluxed for 2 days. The solid was collected *via* filtration, washed repeatedly with anhydrous THF and dried under vacuum to yield [*i*Pr_2_N(CH_2_)_2_mim]N(Tf)_2_ as colorless solvent (17.10 g, 99 %). ^1^H NMR (250 MHz, methylene chloride‐d_2_) δ=8.49 (s, 1H), 7.25 (t, J=1.8 Hz, 1H), 7.15 (t, J=1.8 Hz, 1H), 4.04 (d, J=6.1 Hz, 2H), 3.84 (s, 3H), 2.92 (hept, J=6.6 Hz, 2H), 2.72 (t, J=5.5 Hz, 2H), 0.81 ppm (d, J=6.6 Hz, 12H).

### Synthesis of 1‐(2‐(diisopropylamino) ethyl)‐3‐methyl‐imidazolium bis (trifluoromethane) Sulfonylimide 16

[*i*Pr_2_N(CH_2_)_2_mim]Cl (5.0 g, 17.2 mmol) was suspended in dichloromethane. Sodium hydroxide (0.7 g, 17.2 mmol) dissolved in water and added and the biphasic system was stirred for ten minutes at room temperature. Lithium bis (trifluoromethane) sulfonylimide (5.3 g 18.6 mmol) dissolved in water was added dropwise. The mixture was stirred for one hour at room temperature. The biphasic reaction mixture was extracted three times with CH_2_Cl_2_. The combined organic phases were washed repeatedly with MilliQ‐grade H_2_O until no more chloride anions could be detected. The organic phase was dried over Na_2_SO_4_, filtrated and the solvent was removed. Remaining volatile traces were removed under high vacuum with stirring at 50 °C to yield [*i*Pr_2_N(CH_2_)_2_mim]N(Tf)_2_ **16** as colorless liquid (7.7 g, 88 %). ^1^H NMR (250 MHz, methylene chloride‐d_2_) δ=8.49 (s, 1H), 7.25 (t, J=1.8 Hz, 1H), 7.15 (t, J=1.8 Hz, 1H), 4.04 (d, J=6.1 Hz, 2H), 3.84 (s, 3H), 2.92 (hept, J=6.6 Hz, 2H), 2.72 (t, J=5.5 Hz, 2H), 0.81 ppm (d, J=6.6 Hz, 12H).

### General Procedure for the Hydrogenation of Aldehydes on the Example of 4‐fluorobenzadehyde 11

All hydrogenation reactions were carried out in a Roth steel autoclave using a Tecsis manometer. The glass tube of the steel autoclave was charged with ionic liquid (250 mg) and pre‐catalyst **I** (0.01 mmol) in a glove box and placed into the autoclave. The autoclave was evacuated and flushed with argon three times before 4‐flurobenzaldehyde **11** (2 mmol), DBU (0.1 mmol) and *n*‐heptane (1.5 ml) were added. After flushing three times with hydrogen, the desired hydrogen pressure (10 bar) was established and the reaction was stirred at 25 °C for 60 min. before the pressure was released. The organic and ionic liquid phase was separately analyzed by ^19^F{^1^H} NMR spectroscopy. The absolute amounts of aldehyde and alcohol were determined *via* integration using 10 μl fluorobenzene as external standard, and the yield was calculated accordingly.

In case of product isolation, a different work‐up strategy was followed. After the hydrogen pressure was released, the organic phase was separated and the ionic liquid phase was extracted four more times with diethyl ether (2 ml each). The combined organic layers were filtered over silica and the solvent was evaporated. The obtained crude products were purified *via* flash column chromatography to yield the desired alcohols in spectroscopically pure form.

### Procedure for Catalyst Recycling *via* semi‐continuous Substrate Addition

After running the hydrogenation according to the general procedure given above for 10 minutes, the pressure of the autoclave was released. Fresh 4‐fluorobenzaldehyde **11** (2 mmol) in *n*‐heptane (0.5 ml) was added and the autoclave was flushed with hydrogen three times. The hydrogen pressure was adjusted to 10 bar, and the reaction was run for further 10 min. This procedure was done independently with 2, 3 4 and 5 consecutive addition steps. For each experiment, the overall yield was determined after the final run via ^19^F{^1^H} NMR spectroscopy as described above.

**4‐Fluorobenzylalcohol** (isolated yield 95 %): ^1^H NMR (200 MHz, chloroform‐d) δ=7.37–7.15 (m, 2H), 7.06–6.86 (m, 2H), 4.58 (s, 2H), 1.76 ppm (s, 1H).^19^F NMR (235 MHz, chloroform‐d) δ=−113.4 ppm

**Benzylalcohol** (isolated yield 90 %):^1^H NMR (200 MHz, chloroform‐d) δ=7.40–7.14 (m, 5H), 4.63 (s, 2H), 1.6 ppm (s, 1H).

**4‐Tolylalcohol** (isolated yield 93 %):^1^H NMR (250 MHz, chloroform‐d) δ=7.28 (d, J=8.0 Hz, 2H), 7.20 (d, J=8.4 Hz, 1H), 4.66 (s, 2H), 2.38 (s, 3H), 1.80 ppm (s, 1H).

**4‐Methoxybenzylalcohol** (isolated yield 89 %): ^1^H NMR (250 MHz, chloroform‐d) δ=7.32 (d, J=8.3 Hz, 2H), 6.92 (d, J=7.3 Hz, 2H), 4.64 (s, 2H), 3.85 (s, 3H), 1.62 ppm (s, 1H).

**2‐Hydroxybenzylalcohol** (isolated yield 79 %):^1^H NMR (250 MHz, chloroform‐d) δ=7.23–6.88 (m, 3H), 6.78 (td, J=7.8, 1.0 Hz, 2H), 4.76 ppm (s, 2H).

**4‐Chlorbenzylalcohol** (isolated yield 92 %):^1^H NMR (250 MHz, chloroform‐d) δ=7.34–6.24 (m, 3H), 4.61 (s, 2H), 2.69 ppm (s, 1H).

**Pyridin‐2‐ylmethanol** (isolated yield 87 %): ^1^H NMR (250 MHz, chloroform‐d) δ=8.48 (d, J=4.9 Hz, 1H), 7.62 (td, J=7.7, 1.8 Hz, 1H), 7.28–7.05 (m, 2H), 4.70 (s, 2H), 4.02 ppm (s, 1H).

**Furan‐2‐ylmethanol** (isolated yield 87 %): ^1^H NMR (250 MHz, chloroform‐d) δ=7.42 (d, *J*=2.6 Hz, 1H), 6.53–6.28 (m, 2H), 4.61 (s, 2H), 2.10 ppm (s, 1H).

**Thiophen‐2‐ylmethanol** (isolated yield 89 %): ^1^H NMR (250 MHz, chloroform‐d) δ=7.37–7.27 (m, 1H), 7.02 (ddd, J=8.4, 4.7, 3.4 Hz, 2H), 4.87 (s, 2H), 1.74 ppm (s, 1H).

**(*E*)‐3‐Phenylprop‐2‐en‐1‐ol** (isolated yield 88 %):^1^H NMR (250 MHz, chloroform‐d) δ=7.67–7.08 (m, 5H), 6.76–6.48 (m, 1H), 6.47–6.19 (m, 1H), 4.41–4.16 (m, 2H), 1.80 ppm (s, 1H).

**2,2‐Diphenylethan‐1‐ol** (isolated yield 21 %): ^1^H NMR (250 MHz, chloroform‐d) δ=7.45–7.19 (m, 10H), 4.33–4.14 (m, 3H), 1.6 ppm (s, 1H).

**3‐(Benzo [d][1,3] dioxol‐5‐yl)‐2‐methylpropan‐1‐ol** (isolated yield 91 %): ^1^H NMR (250 MHz, chloroform‐d) δ=6.82–6.57 (m, 3H), 5.94 (s, 2H), 3.60–3.42 (m, 2H), 2.69 (dd, *J*=13.5, 6.3 Hz, 1H), 2.37 (dd, *J*=13.5, 8.0 Hz, 1H), 1.90 (dq, *J*=13.1, 6.5 Hz, 1H), 1.77 (s, 1H), 0.93 ppm (d, *J*=6.7 Hz, 3H).

**Octan‐1‐ol** (isolated yield 34 %): ^1^H NMR (250 MHz, chloroform‐d) δ=3.66 (t, J=6.6 Hz, 3H), 1..68–1.51 (m, 3H), 1.46–1.23 (m, 16 H), 0.9 ppm (t, J=6.7 Hz 3H).

**Cyclohex‐3‐en‐1‐ylmethanol** (isolated yield 28 %): ^1^H NMR (250 MHz, chloroform‐d) δ=5.69 (d, J=2.4 Hz, 2H), 3.55 (dd, J=6.0, 1.7 Hz, 3H), 2.23–2.02 (m, 6H), 1.91–1.66 (m, 4H), 1.38–1.20 ppm (m, 1H).

**3,7‐Dimethylocta‐2,6‐dien‐1‐ol** (isolated yield 45 %): ^1^H NMR (250 MHz, chloroform‐d) δ=5.15–5.05 (m, 1H), 3.75–3.59 (m, 2H), 2.14–1.84 (m, 2H), 1.79–1.58 (m, 6H), 1.58–1.36 (m, 2H), 1.36–1.07 (m, 2H), 0.91 ppm (d, J=6.6 Hz, 3H).

**(*E*)‐3,7‐dimethylocta‐2,6‐dien‐1‐ol** (isolated yield 53 %): ^1^H NMR (250 MHz, chloroform‐d) δ=5.43 (t, J=6.4 Hz, 1H), 5.11 (t, J=6 Hz, 1H), 4.17 (d, J=6.9 Hz, 2H), 2.19–1.99 (m, 5H), 1.7 (s, 6H), 1.62 ppm (s, 3H).

## Conflict of interest

The authors declare no conflict of interest.

## Supporting information

As a service to our authors and readers, this journal provides supporting information supplied by the authors. Such materials are peer reviewed and may be re‐organized for online delivery, but are not copy‐edited or typeset. Technical support issues arising from supporting information (other than missing files) should be addressed to the authors.

Supplementary
