# Supplementary material for: Selective Hydrogenation of Aldehydes Using a Well‐Defined Fe(II) PNP Pincer Complex in Biphasic Medium
Source: ChemCatChem. 2018 Aug 22;10(19):4386–94. doi: 10.1002/cctc.201800841 (PMC6221069; doi:10.1002/cctc.201800841)
Supplement: Supplementary file 1 — Supplementary [file CCTC-10-4386-s001.pdf]

Supporting Information

© Copyright Wiley-VCH Verlag GmbH & Co. KGaA, 69451 Weinheim, 2018

Selective Hydrogenation of Aldehydes Using a Well-Defined Fe(II) PNP Pincer Complex in Biphasic Medium

Stefan Weber, Julian Brünig, Veronika Zeindlhofer, Christian Schröder, Berthold Stöger, Andreas Limbeck, Karl Kirchner,* and Katharina Bica*© 2018 The Authors. Published by Wiley-VCH Verlag GmbH & Co. KGaA. This is an open access article under the terms of the Creative Commons Attribution License, which permits use, distribution and reproduction in any medium, provided the original work is properly cited.

Selective hydrogenation of aldehydes using a well-defined Fe(II) PNP pincer complex in biphasic medium

Stefan Weber,[a] Julian Brünig,[a] Veronika Zeindlhofer,[b] Christian Schröder,[b] Berthold Stöger,[c] Andreas Limbeck,[d] Karl Kirchner*,[a] and Katharina Bica*,[a]

[a] Institute of Applied Synthetic Chemistry, Vienna University of Technology, Getreidemarkt 9/163-AC, A-1060 Wien, Austria. Corresponding authors: Katharina Schröder (born K. Bica) katharina.schroeder@tuwien.ac.at (+43 1 58801163601) or karl.kirchner@tuwien.ac.at (+43 1 58801 163611)

[b] University of Vienna, Faculty of Chemistry, Department of Computational Biological Chemistry, Währingerstrasse 17, A-1090 Wien, Austria.

[c] X-Ray Center, Vienna University of Technology, Getreidemarkt 9, A-1060 Wien, Austria.

[d] Institute of Chemical Technologies and Analytics, Vienna University of Technology, Getreidemarkt 9/163-AC, A-1060 Wien, Austria.

1 Stability of (pre-)catalyst **I** and catalyst **II** in ionic liquid **4**

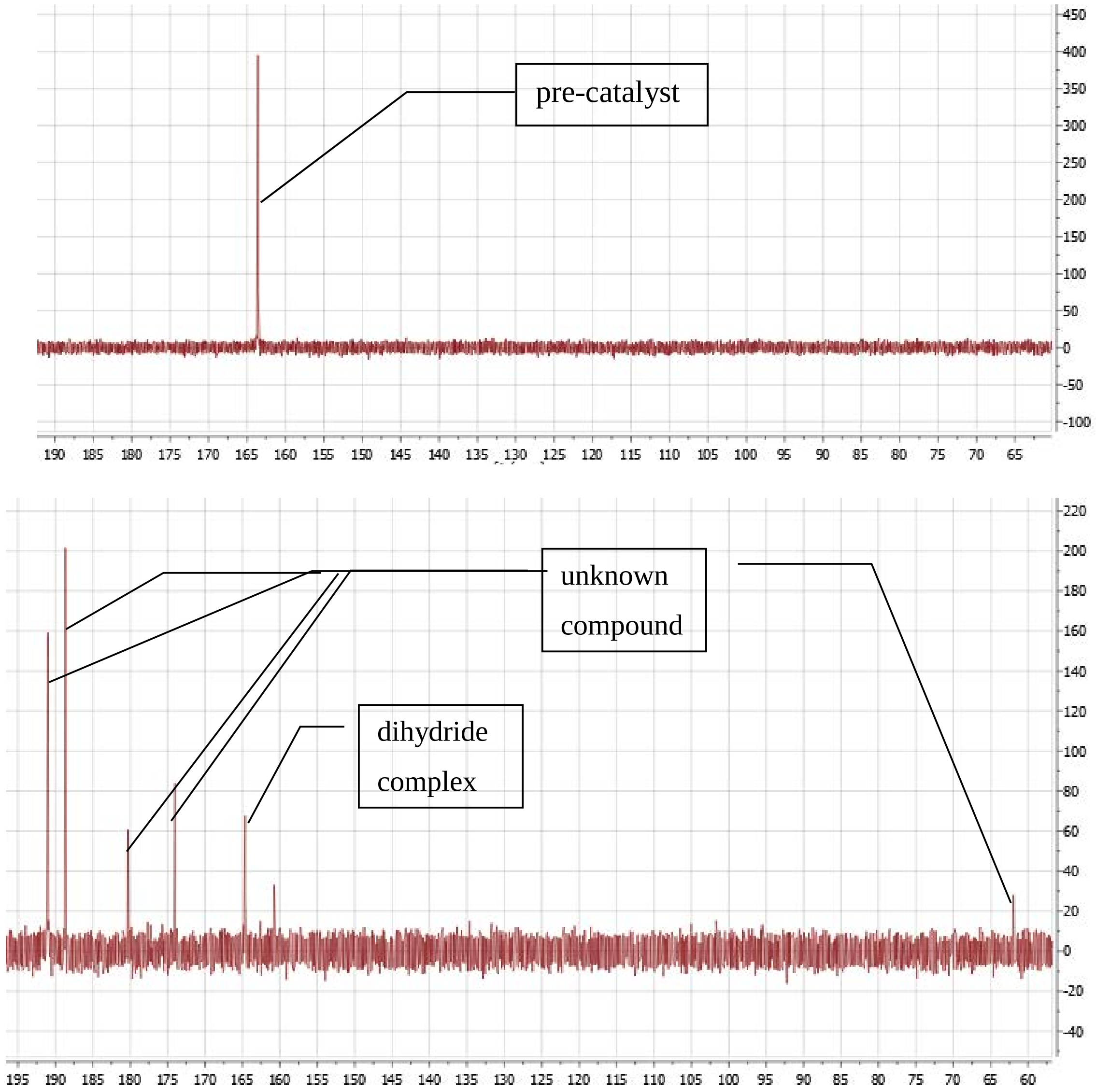

Figure 1. ^{31}*P-NMR of pre-catalyst* ***I*** *in [P$_{4441}$]NTf$_2$ after being dissolved for one week (top),* ^{31}*P-NMR of the dihydride complex* ***II*** *in [P$_{4441}$]NTf$_2$] after being dissolved for two hours (bottom)*

2 Spectra of synthesized $N(Tf)_2$-based Brønsted basic ionic liquids

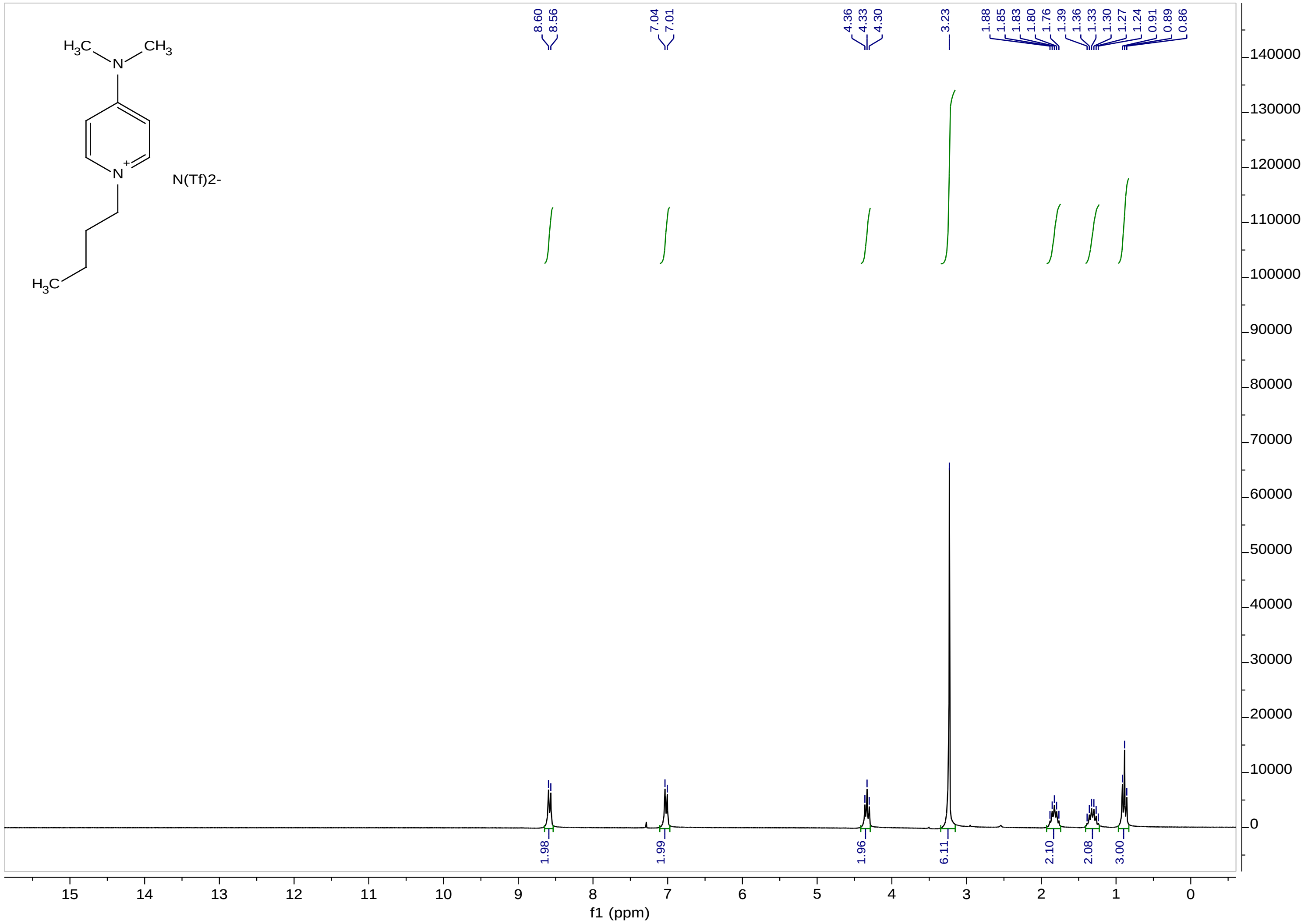

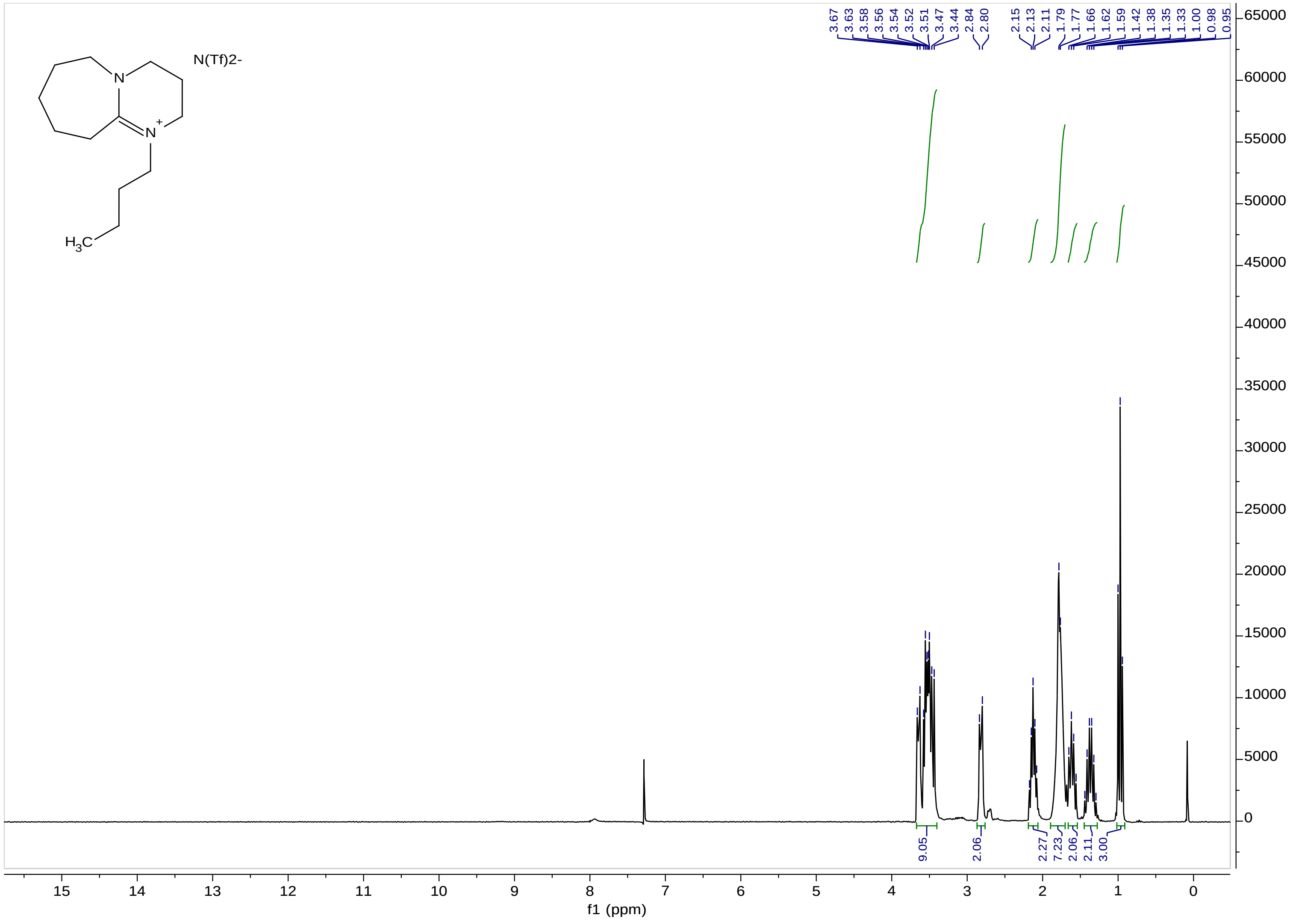

N(Tf)2-
H_3C
3.67 3.63 3.58 3.56 3.54 3.52 3.51 3.47 3.44 2.84 2.80 2.15 2.13 2.11 1.79 1.77 1.66 1.62 1.59 1.42 1.38 1.35 1.33 1.00 0.98 0.95
9.05 2.06 2.27 7.23 2.06 2.11 3.00
65000 60000 55000 50000 45000 40000 35000 30000 25000 20000 15000 10000 5000 0
15 14 13 12 11 10 9 8 7 6 5 4 3 2 1 0
f1 (ppm)

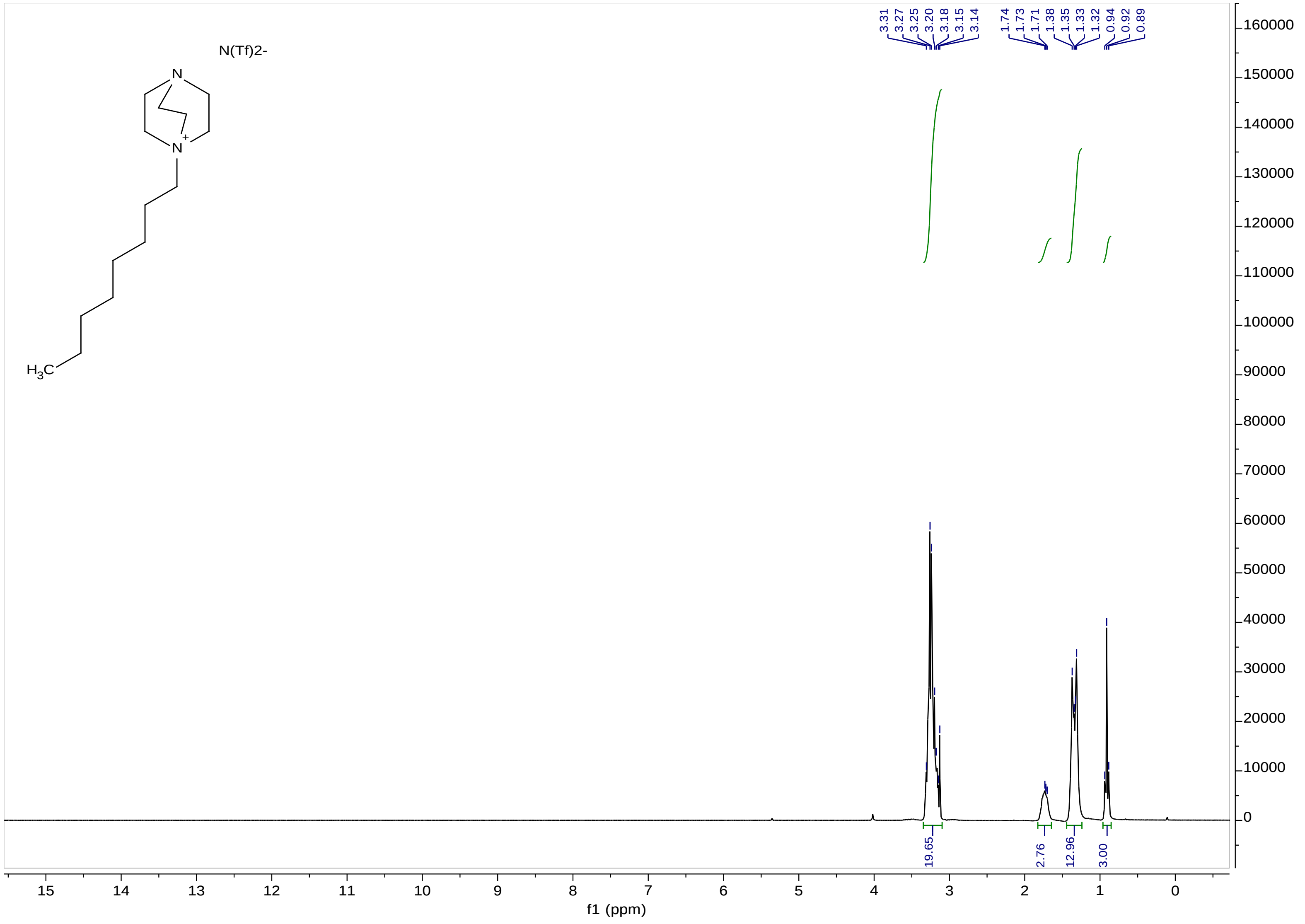

N(Tf)2-
N
N+
H3C
3.31
3.27
3.25
3.20
3.18
3.15
3.14
1.74
1.73
1.71
1.38
1.35
1.33
1.32
0.94
0.92
0.89
19.65
2.76
12.96
3.00
f1 (ppm)

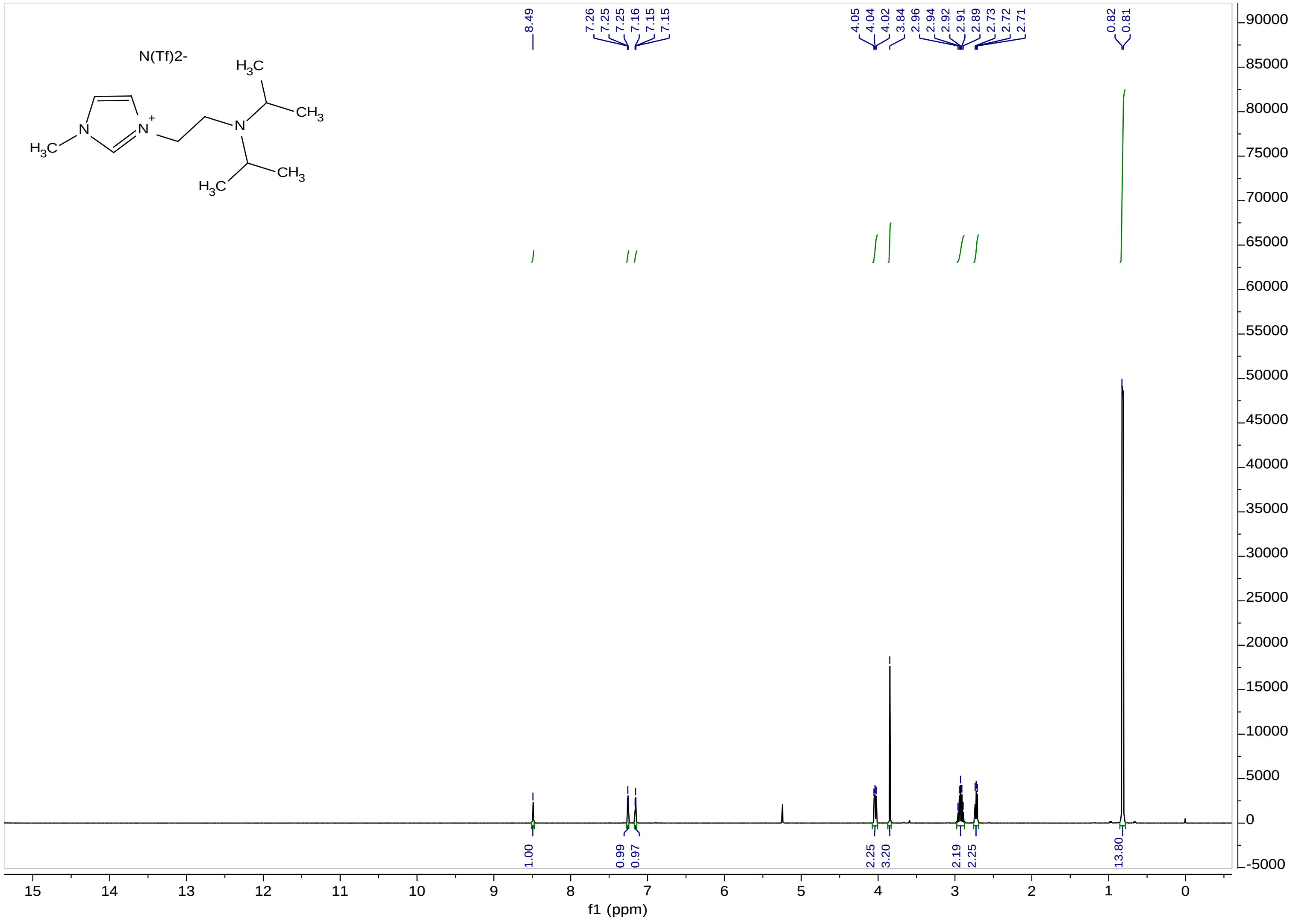
N(Tf)2-
8.49
7.26 7.25 7.25 7.16 7.15 7.15
4.05 4.04 4.02 3.84 2.96 2.94 2.92 2.91 2.89 2.73 2.72 2.71
0.82 0.81
1.00
0.99 0.97
2.25 3.20
2.19 2.25
13.80
f1 (ppm)

3 Spectra of synthesized products

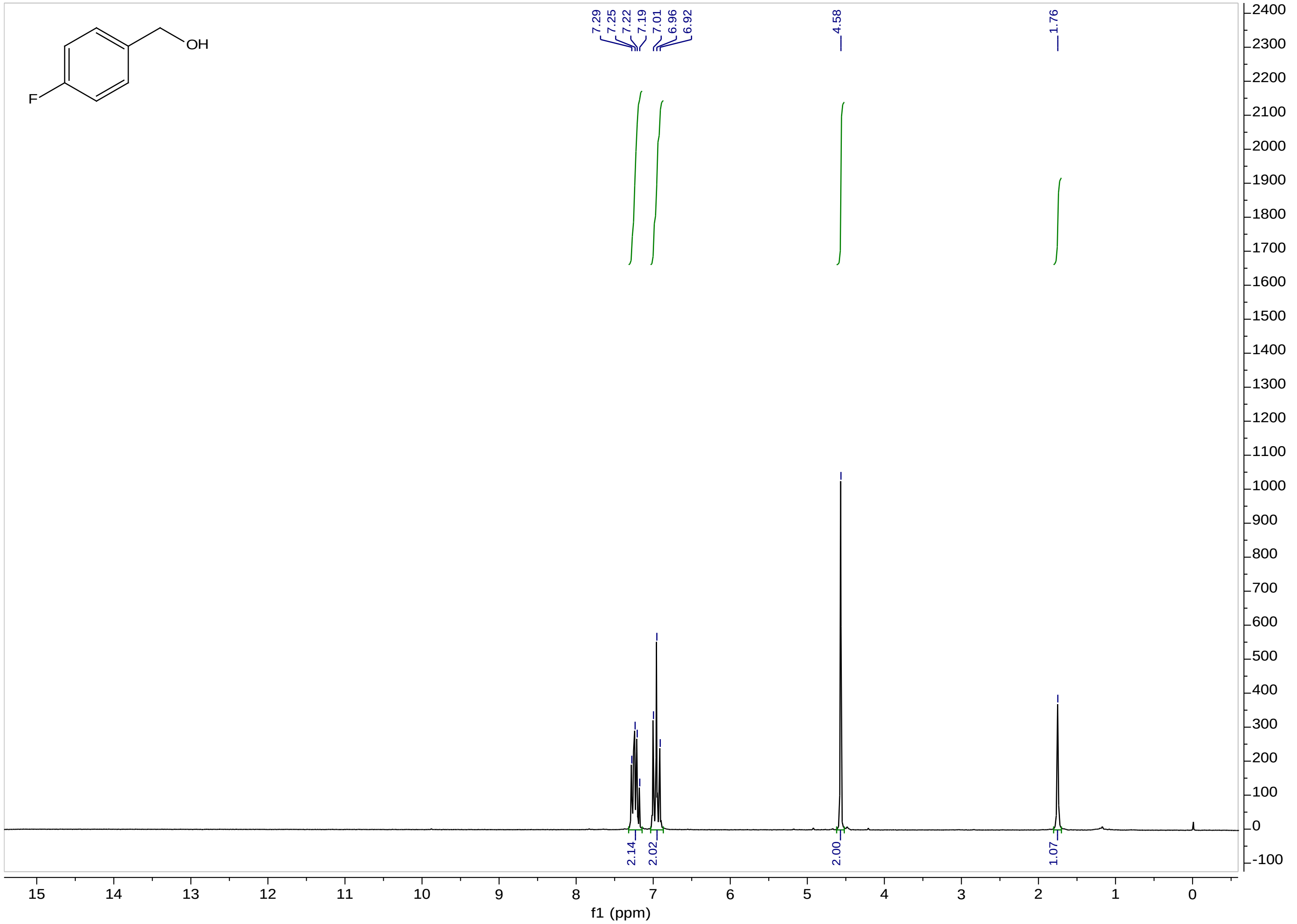

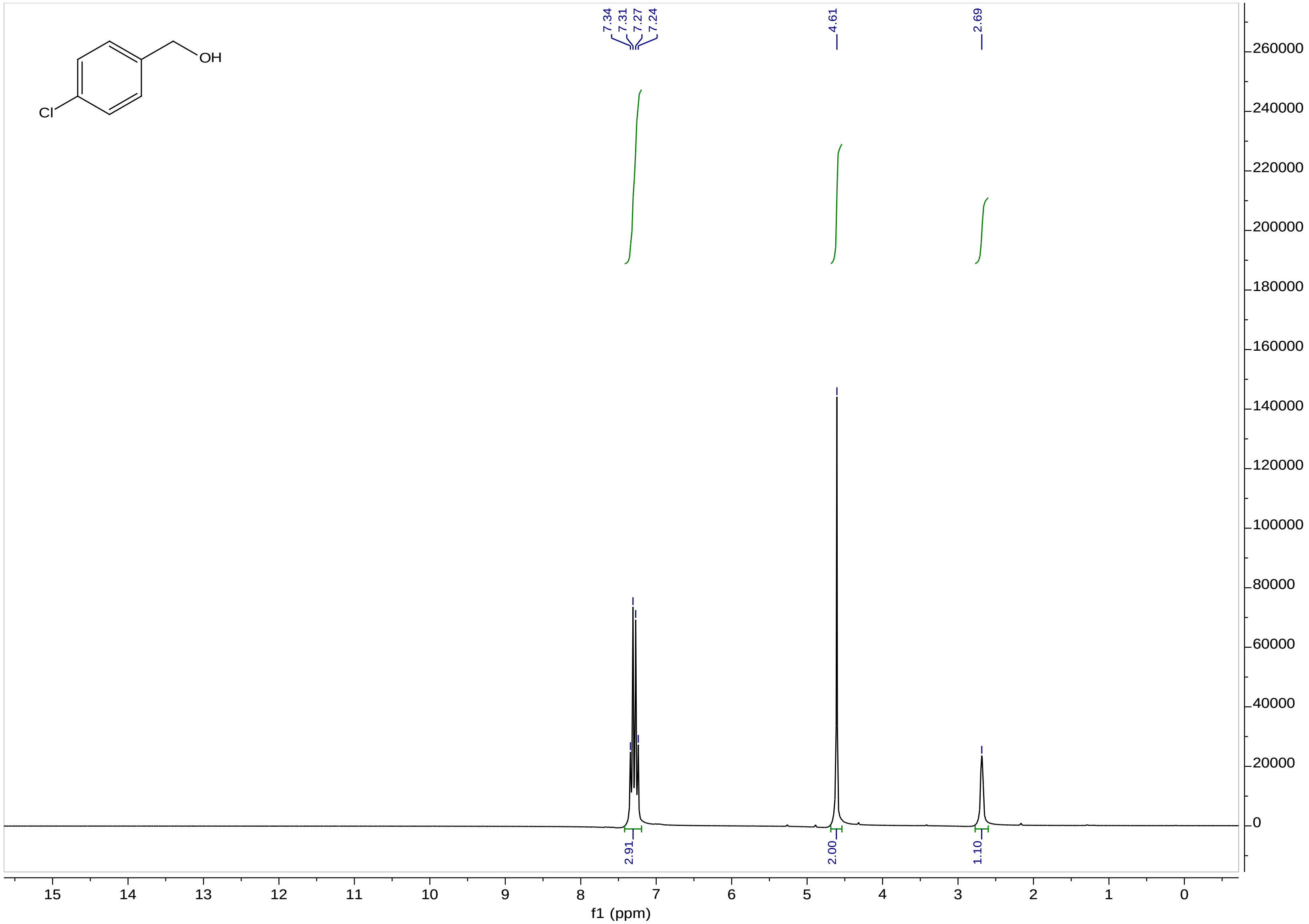

OH
Cl
7.34
7.31
7.27
7.24
4.61
2.69
2.91
2.00
1.10
260000
240000
220000
200000
180000
160000
140000
120000
100000
80000
60000
40000
20000
0
15
14
13
12
11
10
9
8
7
6
5
4
3
2
1
0
f1 (ppm)

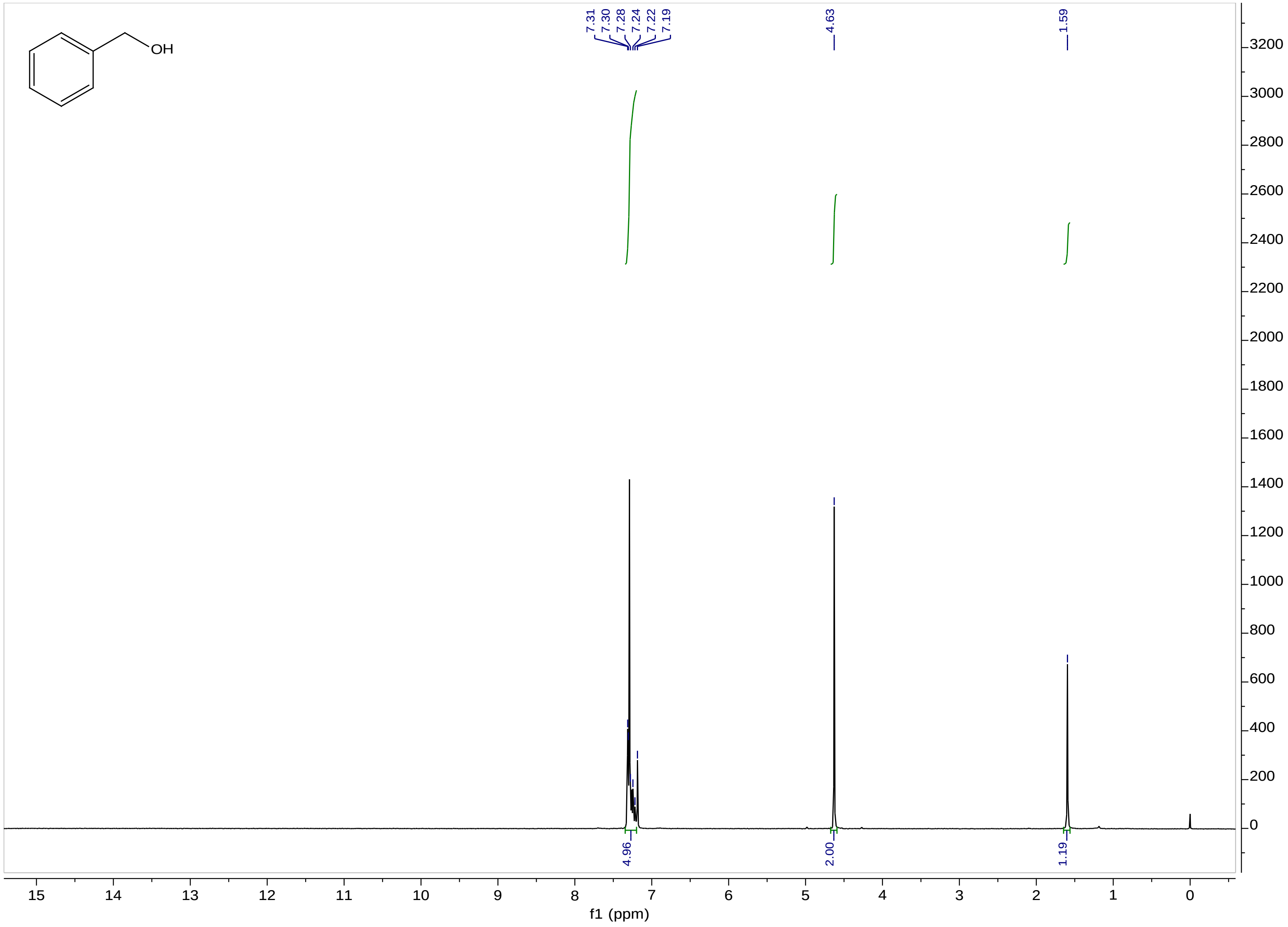

OH
7.31
7.30
7.28
7.24
7.22
7.19
4.63
1.59
4.96
2.00
1.19
f1 (ppm)

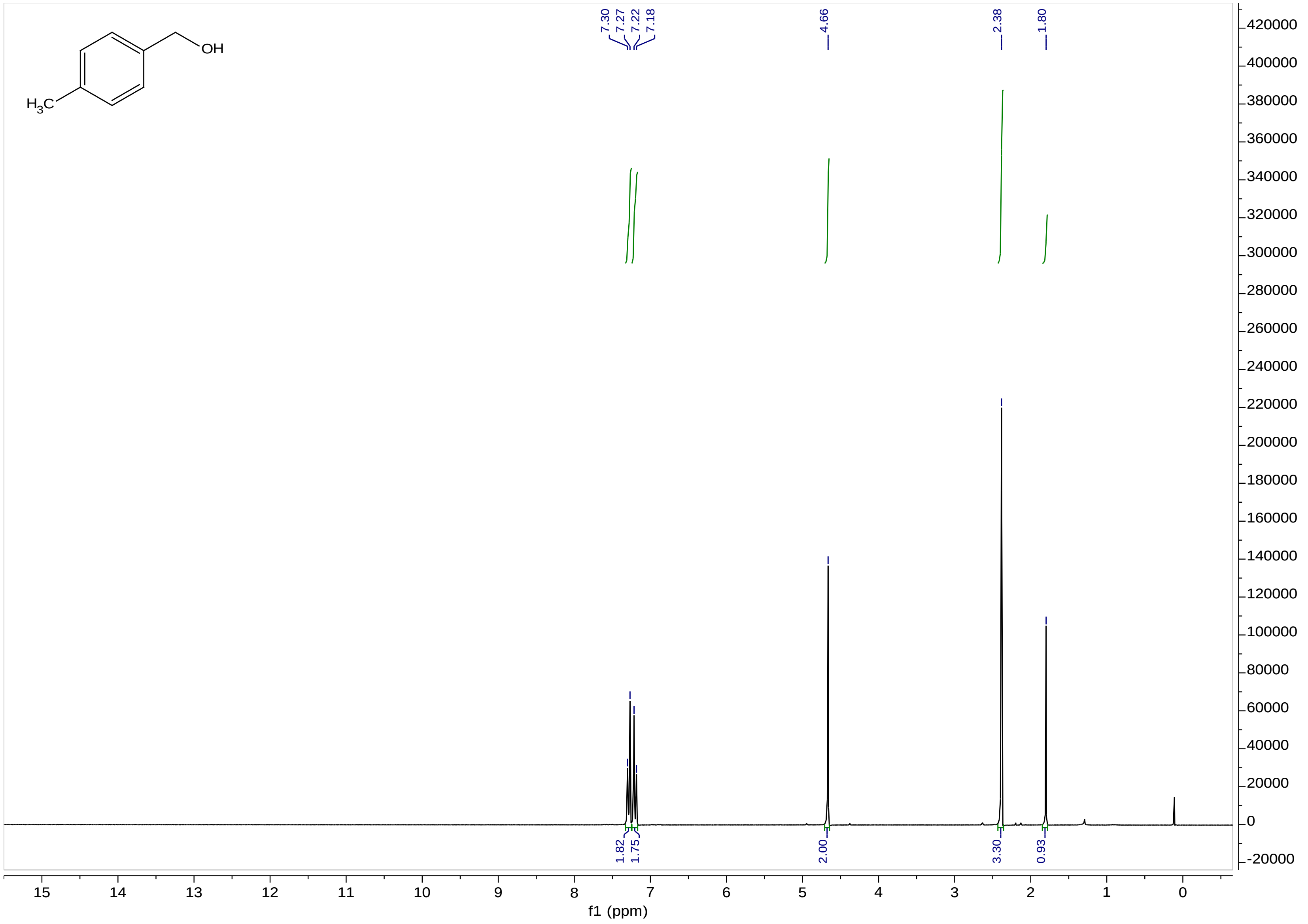

OH
H3C
7.30
7.27
7.22
7.18
4.66
2.38
1.80
1.82
1.75
2.00
3.30
0.93
420000
400000
380000
360000
340000
320000
300000
280000
260000
240000
220000
200000
180000
160000
140000
120000
100000
80000
60000
40000
20000
0
-20000
15
14
13
12
11
10
9
8
7
6
5
4
3
2
1
0
f1 (ppm)

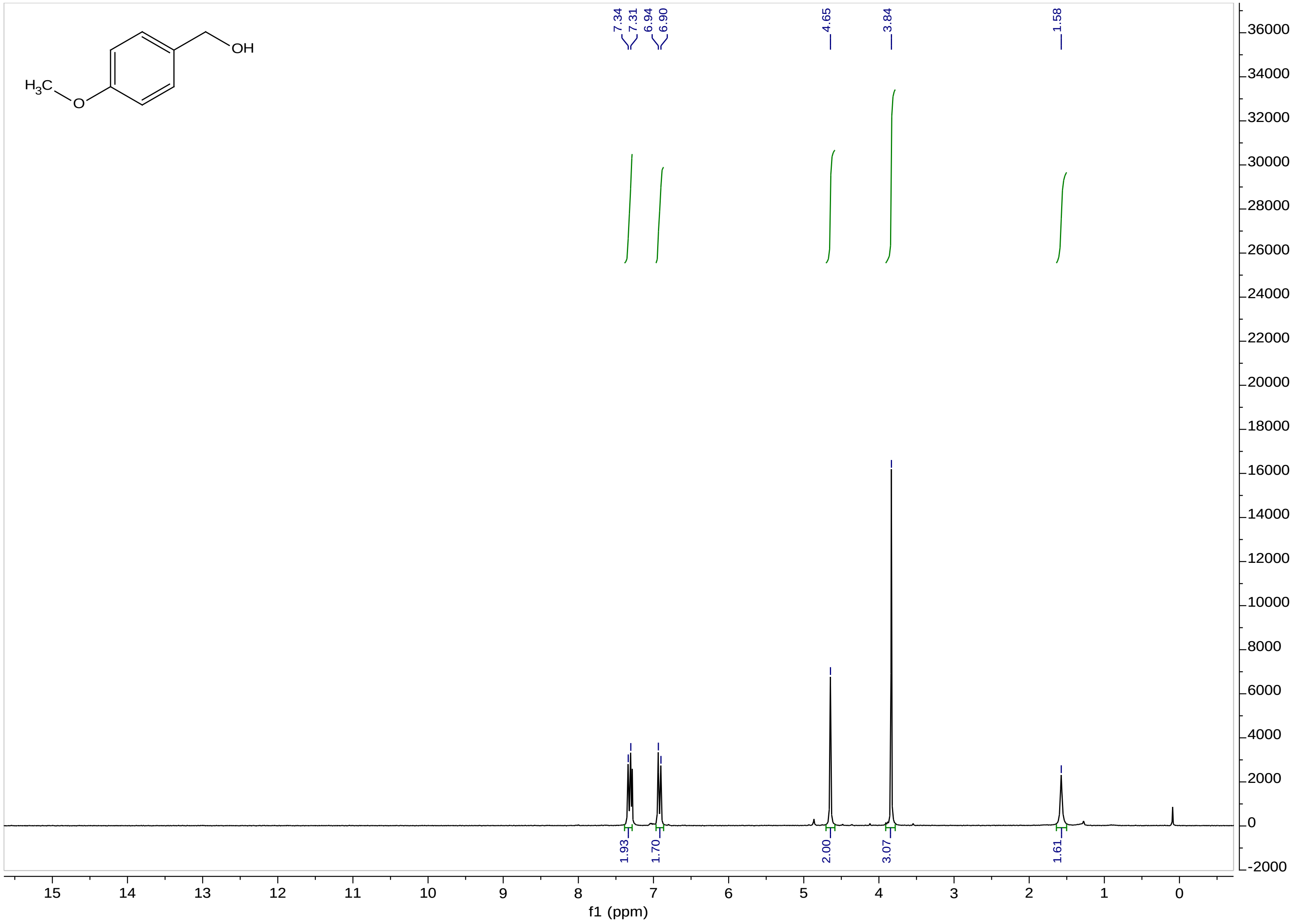
H3C
O
OH
7.34
7.31
6.94
6.90
4.65
3.84
1.58
1.93
1.70
2.00
3.07
1.61
36000
34000
32000
30000
28000
26000
24000
22000
20000
18000
16000
14000
12000
10000
8000
6000
4000
2000
0
-2000
15
14
13
12
11
10
9
8
7
6
5
4
3
2
1
0
f1 (ppm)

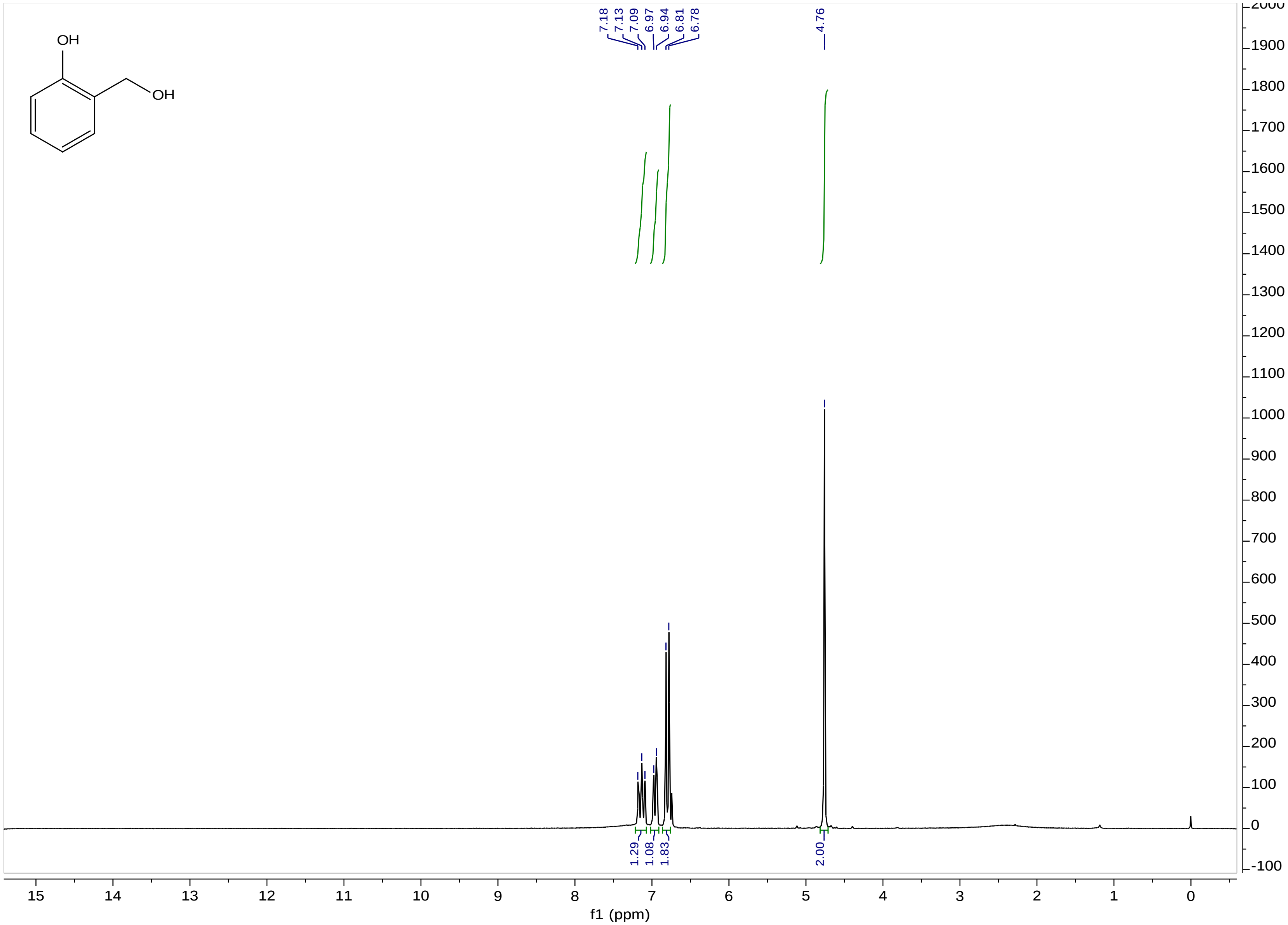
OH
OH
7.18
7.13
7.09
6.97
6.94
6.81
6.78
4.76
1.29
1.08
1.83
2.00
15
14
13
12
11
10
9
8
7
6
5
4
3
2
1
0
f1 (ppm)
2000
1900
1800
1700
1600
1500
1400
1300
1200
1100
1000
900
800
700
600
500
400
300
200
100
0
-100

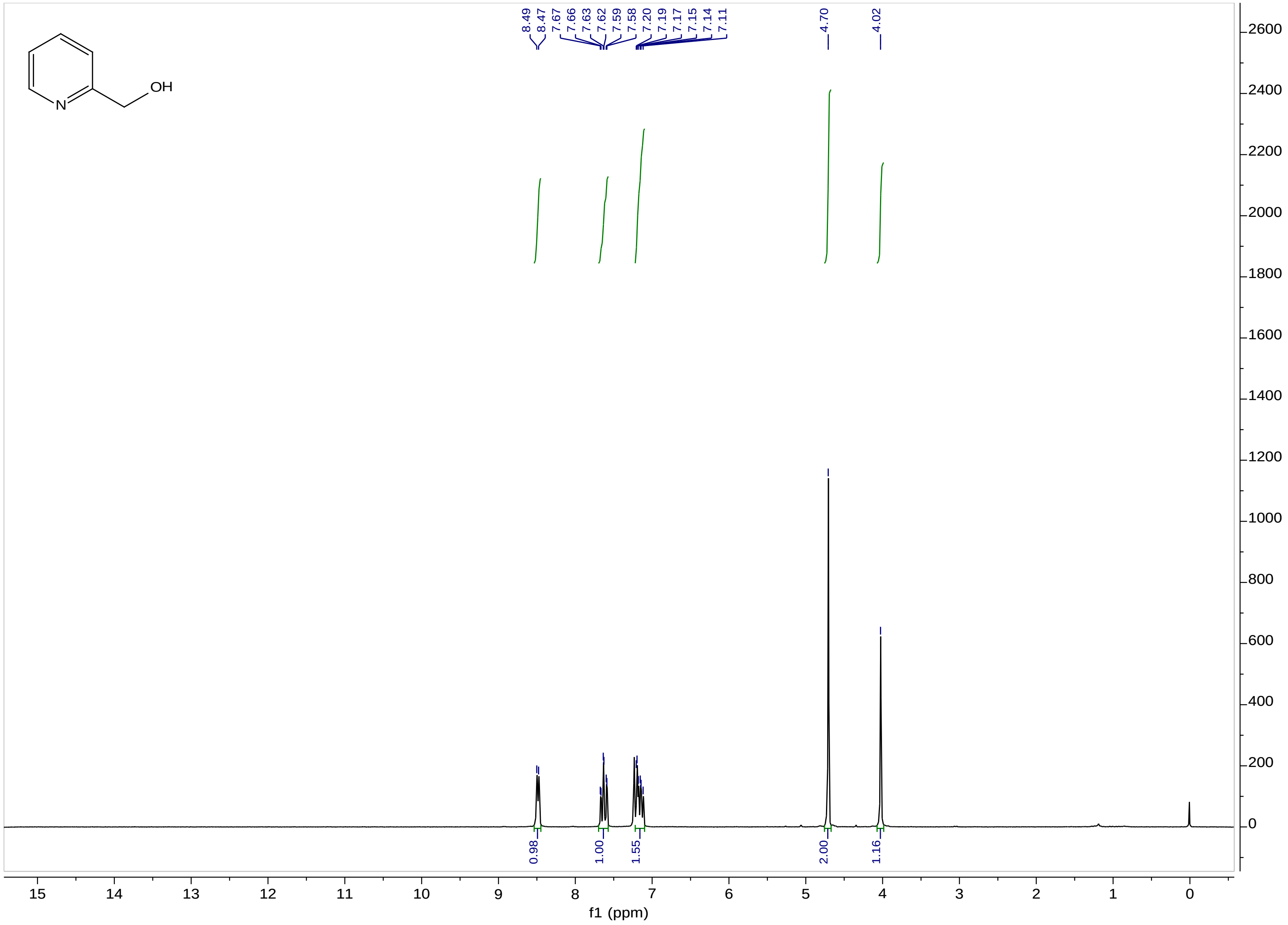

OH
N
8.49
8.47
7.67
7.66
7.63
7.62
7.59
7.58
7.20
7.19
7.17
7.15
7.14
7.11
4.70
4.02
0.98
1.00
1.55
2.00
1.16
2600
2400
2200
2000
1800
1600
1400
1200
1000
800
600
400
200
0
15
14
13
12
11
10
9
8
7
6
5
4
3
2
1
0
f1 (ppm)

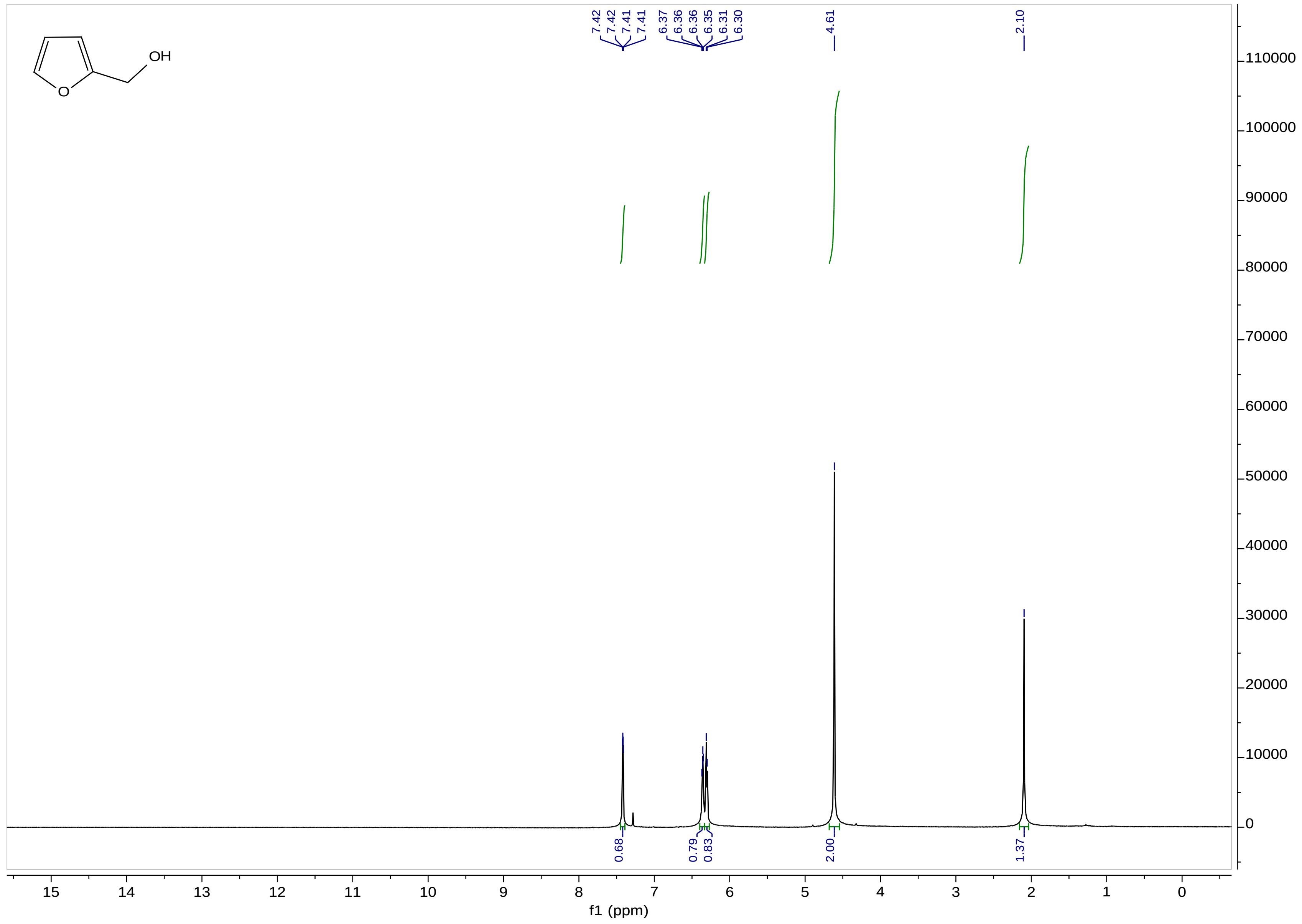
OH
O
7.42
7.42
7.41
7.41
6.37
6.36
6.36
6.35
6.31
6.30
4.61
2.10
0.68
0.79
0.83
2.00
1.37
110000
100000
90000
80000
70000
60000
50000
40000
30000
20000
10000
0
15 14 13 12 11 10 9 8 7 6 5 4 3 2 1 0
f1 (ppm)

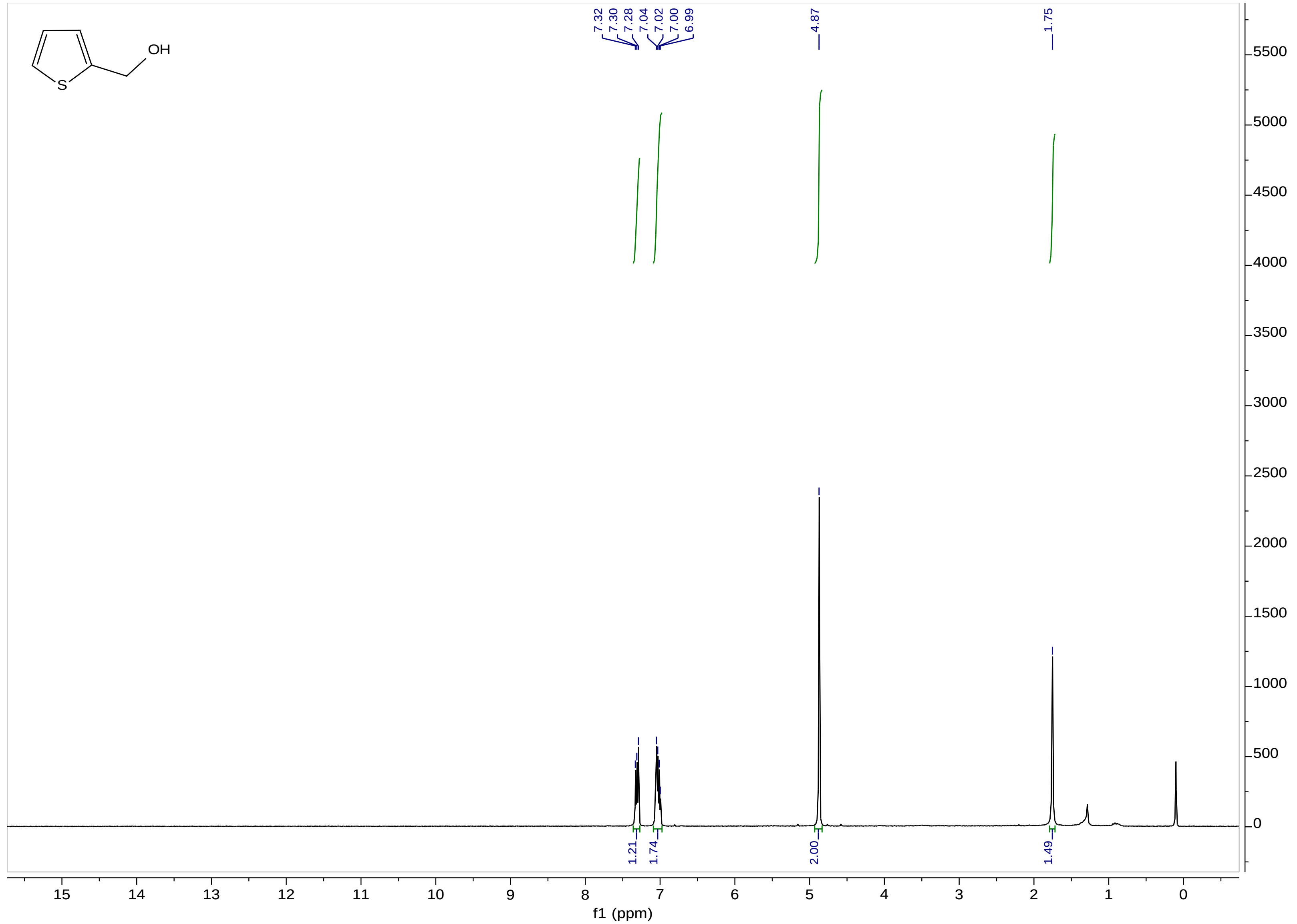

OH
S
7.32
7.30
7.28
7.04
7.02
7.00
6.99
4.87
1.75
1.21
1.74
2.00
1.49
f1 (ppm)

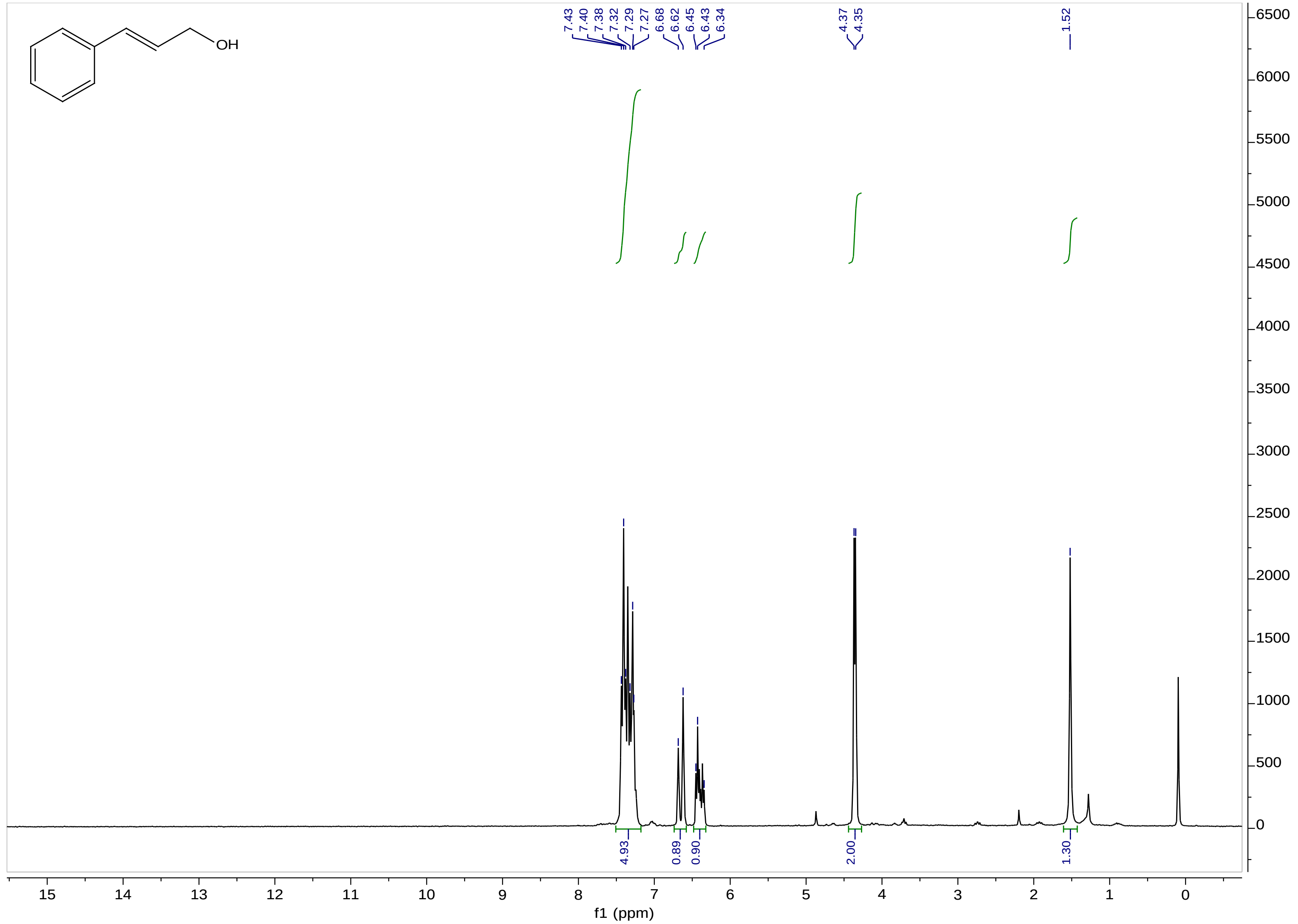

OH
7.43
7.40
7.38
7.32
7.29
7.27
6.68
6.62
6.45
6.43
6.34
4.37
4.35
1.52
4.93
0.89
0.90
2.00
1.30
f1 (ppm)

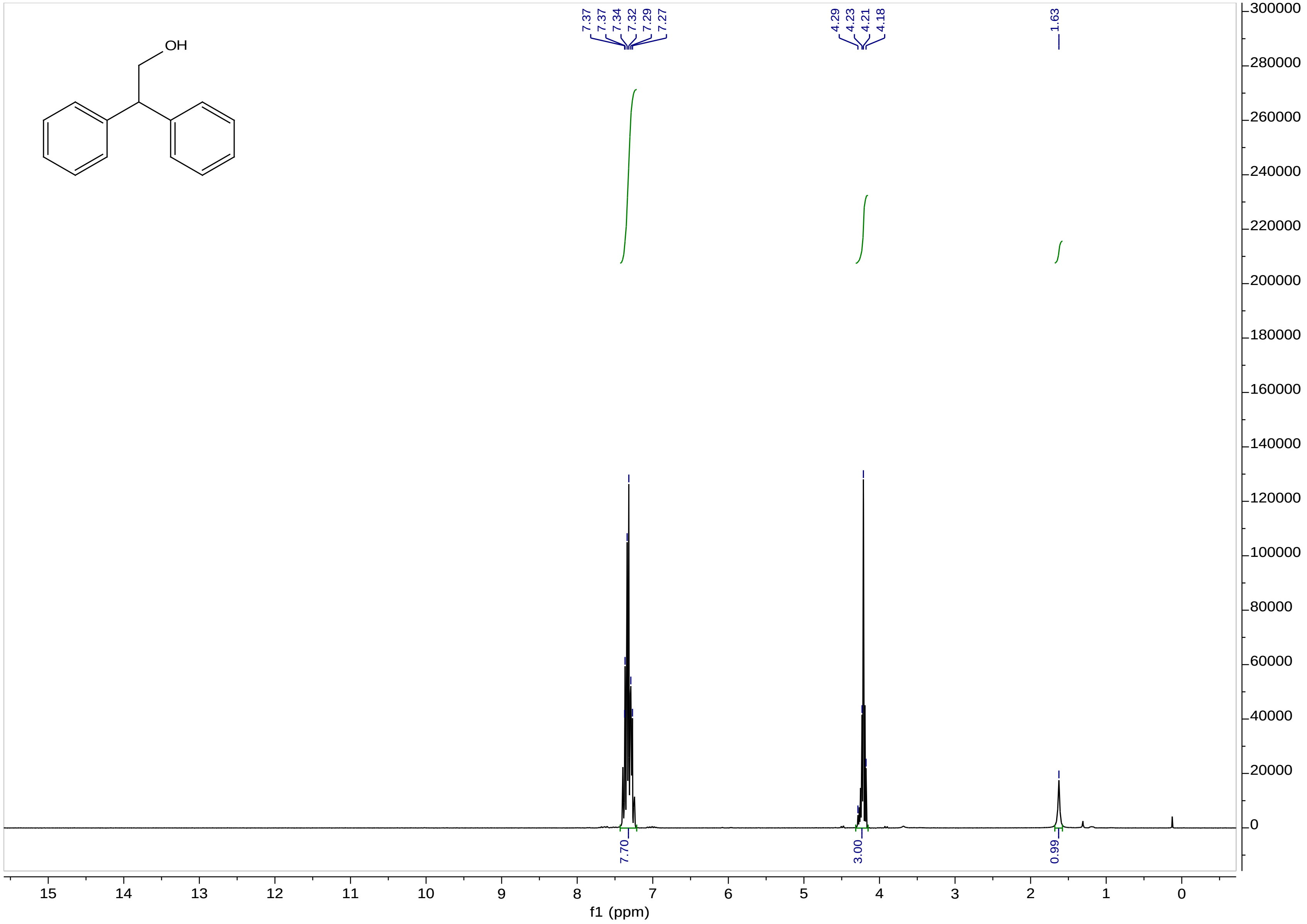

OH
7.37
7.37
7.34
7.32
7.29
7.27
4.29
4.23
4.21
4.18
1.63
7.70
3.00
0.99
300000
280000
260000
240000
220000
200000
180000
160000
140000
120000
100000
80000
60000
40000
20000
0
15
14
13
12
11
10
9
8
7
6
5
4
3
2
1
0
f1 (ppm)

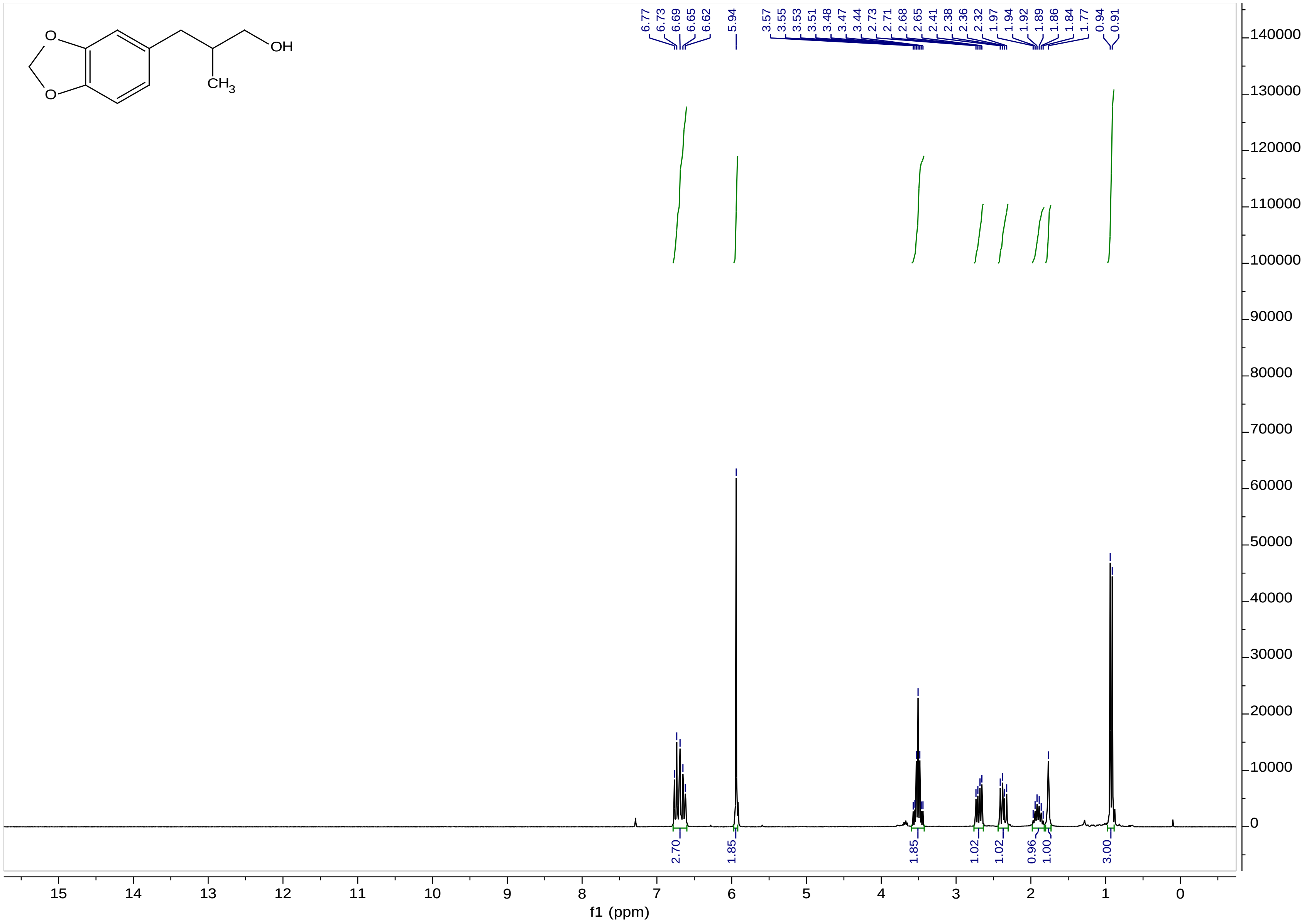
OH
CH3
O
O
6.77
6.73
6.69
6.65
6.62
5.94
3.57
3.55
3.53
3.51
3.48
3.47
3.44
2.73
2.71
2.68
2.65
2.41
2.38
2.36
2.32
1.97
1.94
1.92
1.89
1.86
1.84
1.77
0.94
0.91
2.70
1.85
1.85
1.02
1.02
0.96
1.00
3.00
f1 (ppm)

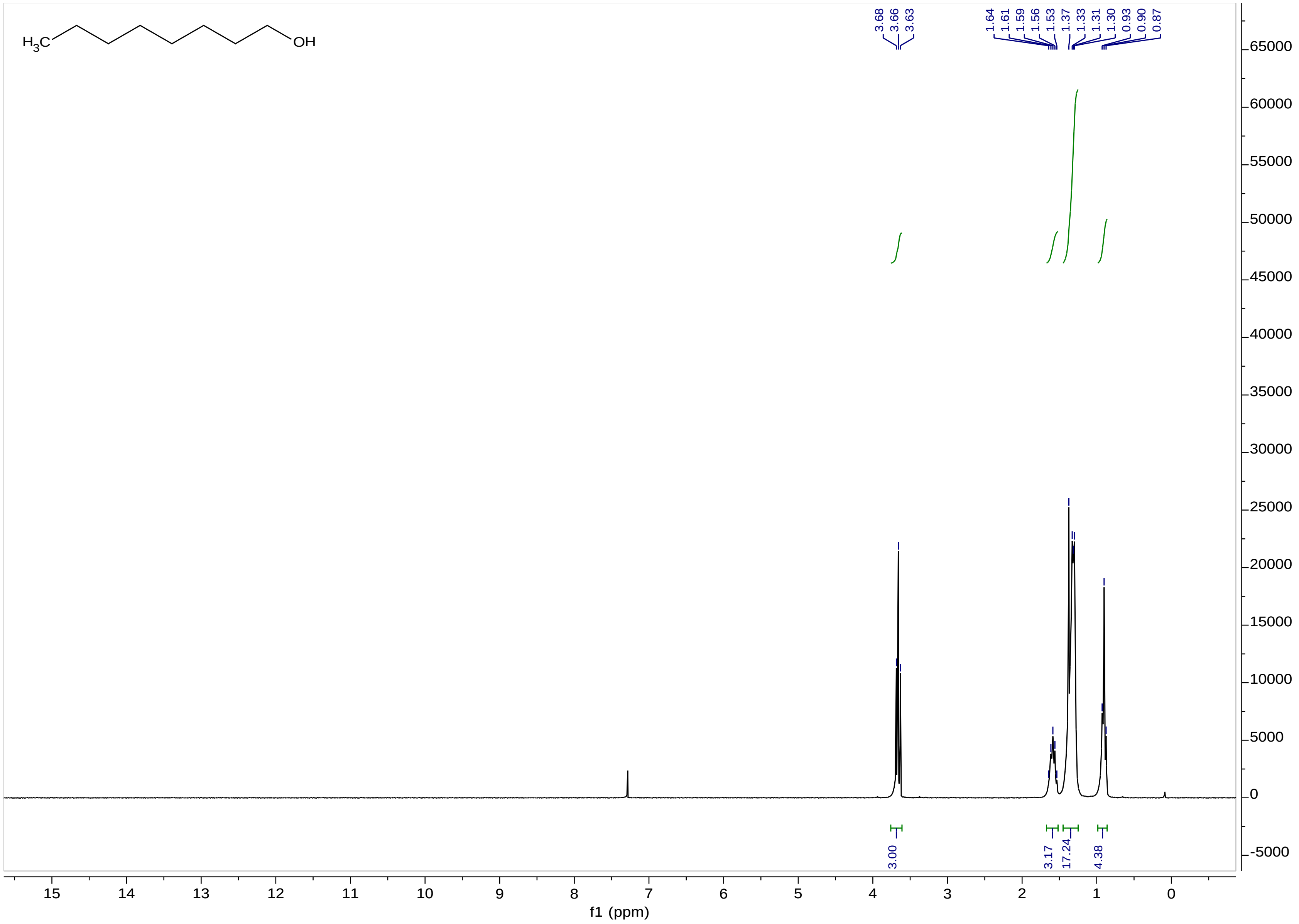

H3C
OH
3.68
3.66
3.63
1.64
1.61
1.59
1.56
1.53
1.37
1.33
1.31
1.30
0.93
0.90
0.87
3.00
3.17
17.24
4.38
f1 (ppm)

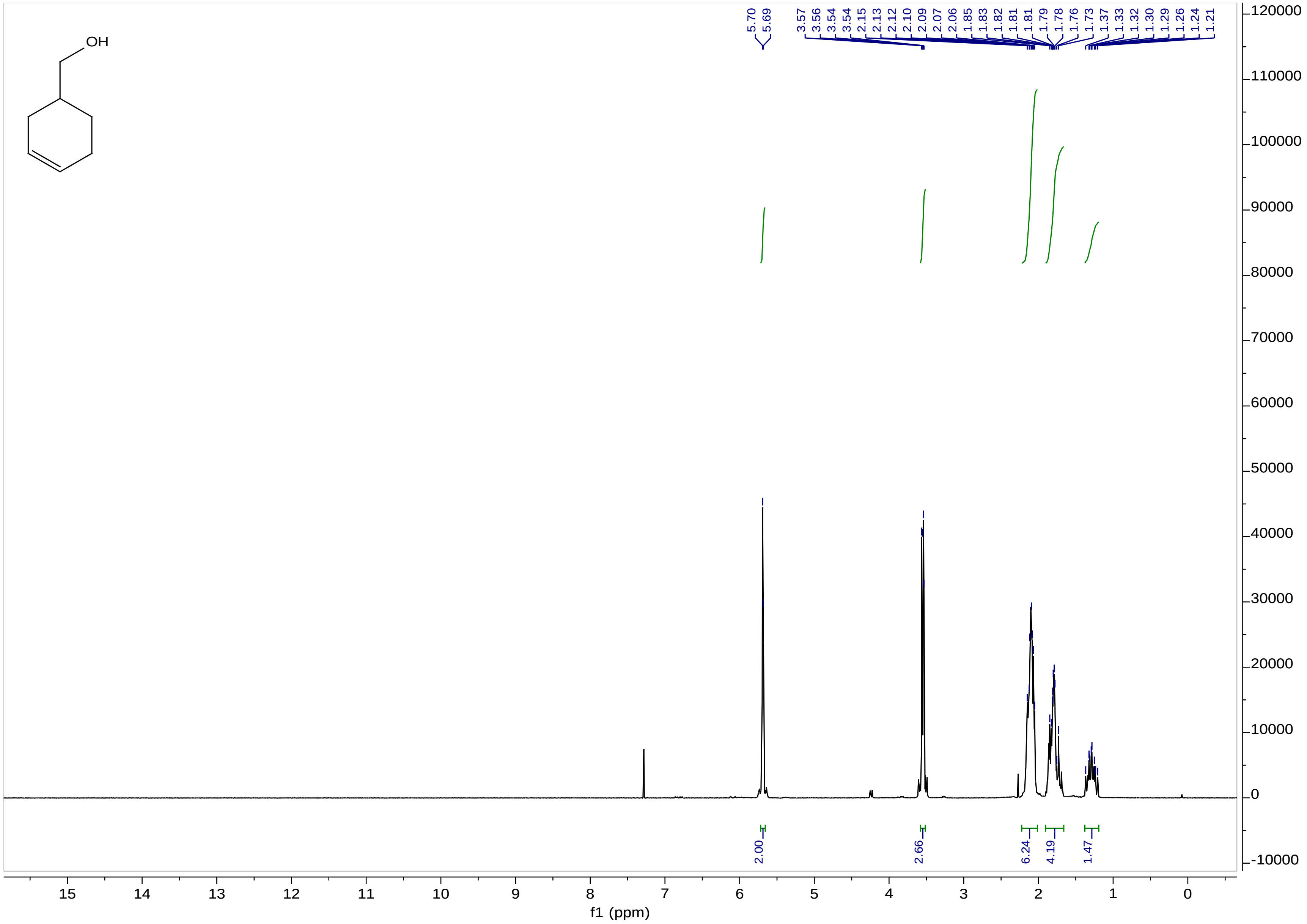
OH
5.70
5.69
3.57
3.56
3.54
3.54
2.15
2.13
2.12
2.10
2.09
2.07
2.06
1.85
1.83
1.82
1.81
1.81
1.79
1.78
1.76
1.73
1.37
1.33
1.32
1.30
1.29
1.26
1.24
1.21
2.00
2.66
6.24
4.19
1.47
f1 (ppm)

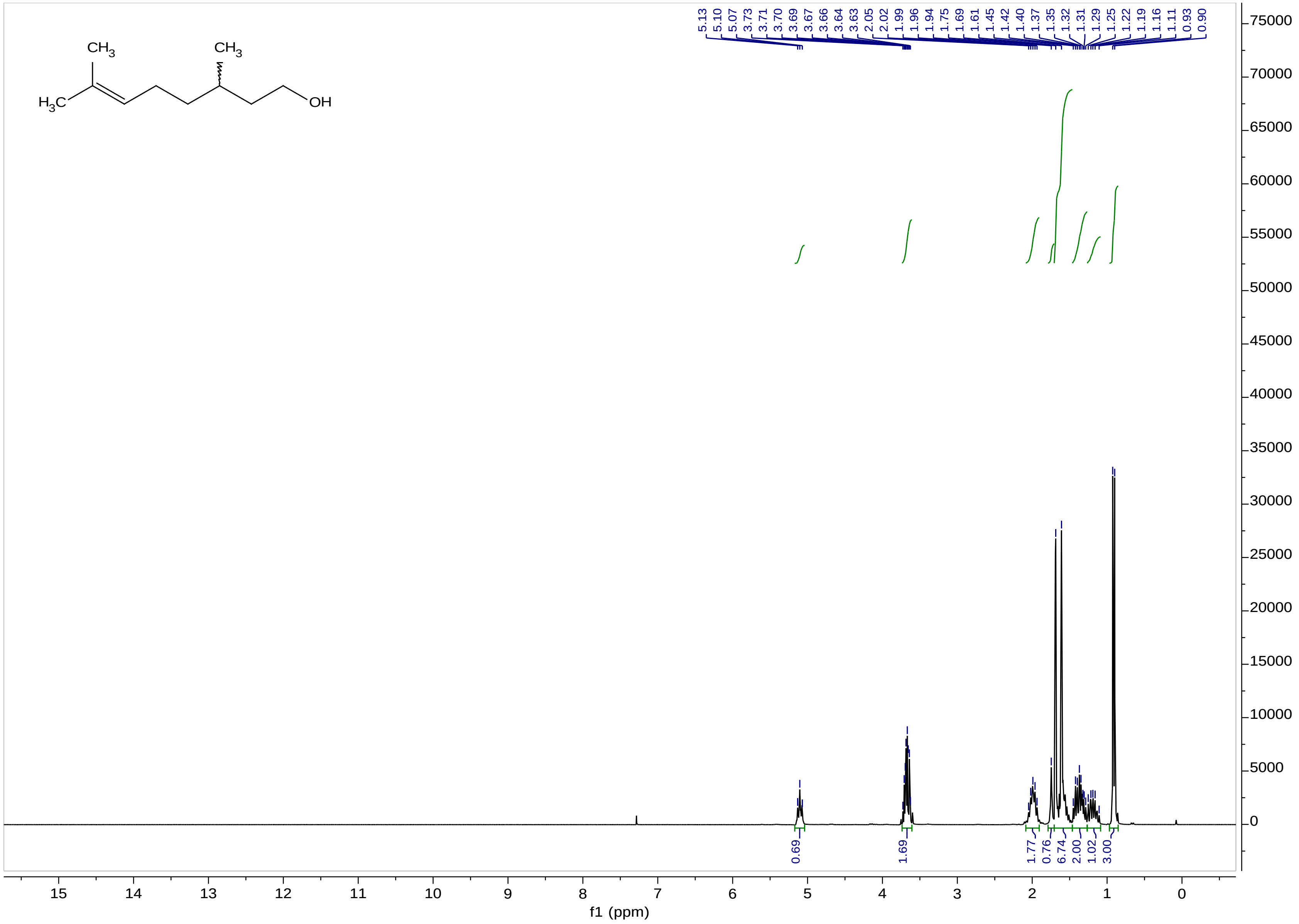

CH3
CH3
H3C
OH
5.13
5.10
5.07
3.73
3.71
3.70
3.69
3.67
3.66
3.64
3.63
2.05
2.02
1.99
1.96
1.94
1.75
1.69
1.61
1.45
1.42
1.40
1.37
1.35
1.32
1.31
1.29
1.25
1.22
1.19
1.16
1.11
0.93
0.90
0.69
1.69
1.77
0.76
6.74
2.00
1.02
3.00
f1 (ppm)

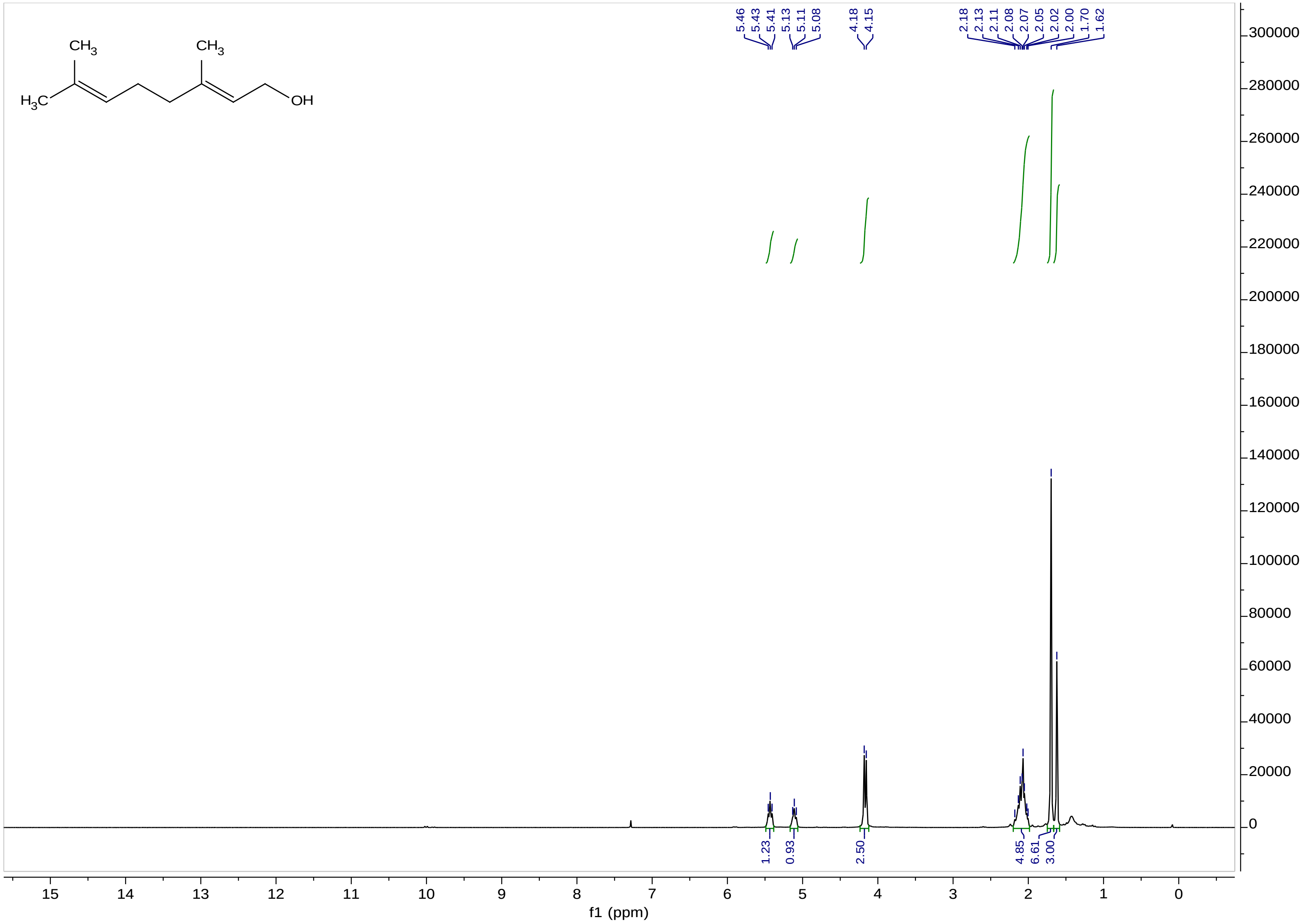
CH3
CH3
H3C
OH
5.46
5.43
5.41
5.13
5.11
5.08
4.18
4.15
2.18
2.13
2.11
2.08
2.07
2.05
2.02
2.00
1.70
1.62
1.23
0.93
2.50
4.85
6.61
3.00
300000
280000
260000
240000
220000
200000
180000
160000
140000
120000
100000
80000
60000
40000
20000
0
15
14
13
12
11
10
9
8
7
6
5
4
3
2
1
0
f1 (ppm)

4 Crystal structure determination

Table 1. Details for the crystal structure determinations of $[C_4DMAP]Cl{\cdot}CHCl_3$.

	$[C_4DMAP]Cl{\cdot}CHCl_3$
formula	$C_{12}H_{20}Cl_4N_2$
fw	334.1
cryst.size, mm	0.54 x 0.21 x 0.04
color, shape	colourless, block
crystal system	orthorhombic
space group	*Pbcn* (no. 60)
a, Å	11.3793(3)
b, Å	11.5408(4)
c, Å	26.4568(9)
V, Å^3	3474.47(19)
T, K	150
Z, Z'	2, 1
ρ_{calc}, g cm^{-3}	1.2775
μ, mm^{-1} (MoKα)	0.668
F(000)	1392
absorption	multi-scan
T_{min}-T_{max}	0.65–0.85
θ range, deg	1.54–30.14
no. of rflns measd	29632
R_{int}	0.0573
no. of rflns unique	5123
no. of rflns	3541
no. of params /	204 / 0
R ($I > 3\sigma(I)$) [a]	0.0448
R (all data)	0.0706
wR ($I > 3\sigma(I)$)	0.1118
wR (all data)	0.1219
GooF	1.49
Diff.Four.peaks	-0.42 / 0.40
CCDC no.	1825413

[a] $R = \Sigma||F_o|-|F_c|| / \Sigma|F_o|$, $wR = \Sigma w(|F_o| - |F_c|) / \Sigma w|F_o|$, GooF = $\{\Sigma[w(F_o^2 - F_c^2)^2] / (n-p)\}^{½}$
